# Single-cell RNA sequencing reveals the dynamics and heterogeneity of lymph node immune cells during acute and chronic viral infections

**DOI:** 10.3389/fimmu.2024.1341985

**Published:** 2024-01-29

**Authors:** Yubei Jin, Yudan He, Bing Liu, Xiaohui Zhang, Caimei Song, Yunchen Wu, Wenjing Hu, Yiwen Yan, Nuo Chen, Yingying Ding, Yuanyuan Ou, Yixiu Wu, Mingxia Zhang, Shaojun Xing

**Affiliations:** ^1^ Guangdong Provincial Key Laboratory of Regional Immunity and Diseases, Department of Pathogen Biology, School of Basic Medical Sciences, Shenzhen University Medical School, Shenzhen University, Shenzhen, China; ^2^ School of Pharmacy, Shenzhen University Medical School, Shenzhen University, Shenzhen, Guangdong, China; ^3^ Department of Life Sciences, Bengbu Medical College, Bengbu, Anhui, China; ^4^ Institute for Hepatology, National Clinical Research Center for Infectious Disease, The Third People’s Hospital of Shenzhen, Shenzhen, Guangdong, China

**Keywords:** chronic viral infection, acute viral infection, ScRNA-seq, scBCR-seq, scTCR-seq, immune landscape

## Abstract

**Introduction:**

The host immune response determines the differential outcome of acute or chronic viral infections. The comprehensive comparison of lymphoid tissue immune cells at the single-cell level between acute and chronic viral infections is largely insufficient.

**Methods:**

To explore the landscape of immune responses to acute and chronic viral infections, single-cell RNA sequencing(scRNA-seq), scTCR-seq and scBCR-seq were utilized to evaluate the longitudinal dynamics and heterogeneity of lymph node CD45^+^ immune cells in mouse models of acute (LCMV Armstrong) and chronic (LCMV clone 13) viral infections.

**Results:**

In contrast with acute viral infection, chronic viral infection distinctly induced more robust NK cells and plasma cells at the early stage (Day 4 post-infection) and acute stage (Day 8 post-infection), respectively. Moreover, chronic viral infection exerted decreased but aberrantly activated plasmacytoid dendritic cells (pDCs) at the acute phase. Simultaneously, there were significantly increased IgA^+^ plasma cells (MALT B cells) but differential usage of B-cell receptors in chronic infection. In terms of T-cell responses, Gzma-high effector-like CD8^+^ T cells were significantly induced at the early stage in chronic infection, which showed temporally reversed gene expression throughout viral infection and the differential usage of the most dominant TCR clonotype. Chronic infection also induced more robust CD4^+^ T cell responses, including follicular helper T cells (Tfh) and regulatory T cells (Treg). In addition, chronic infection compromised the TCR diversity in both CD8^+^ and CD4^+^ T cells.

**Discussion:**

In conclusion, gene expression and TCR/BCR immune repertoire profiling at the single-cell level in this study provide new insights into the dynamic and differential immune responses to acute and chronic viral infections.

## Introduction

The host immune response determines the outcome of viral infection. In contrast to acute viral infections, persistent infections last a long time when the immune system fails to clear the primary infection (1). Several viruses could establish chronic infections in hosts, such as human immunodeficiency virus type 1 (HIV-1), hepatitis B virus (HBV), and hepatitis C virus (HCV) in humans, and lymphocytic choriomeningitis virus (LCMV) in mice, respectively (1). The establishment of chronic infections results in continuous stimulation of both the innate and adaptive immune cells, which causes sustained alterations of the host immune system (2). The persistent stimulation by viruses exerts a detrimental burden on the immune system and leads to causative diseases in the host (1). Thus, it is essential to explore the systemic alterations of host immune response to chronic infection and restore the function of the immune system to fight against pathogens and causative diseases. Comparative analyses in various aspects of the host immune system have been performed to clarify the difference in immune responses between acute and chronic viral infections (2–6). However, systemic comparison in lymphoid tissue immune cells between acute and chronic viral infections is poorly understood.

The LCMV strains Armstrong (Arm) and clone 13 (Cl13) could induce acute and chronic infections in immunocompetent mice, respectively (1, 7, 8). Therefore, LCMV has been extensively used to explore immune responses against acute and persistent viral infections (9–11). Indeed, the LCMV infection mice model remains an active and productive platform for immunological studies (8, 12). Recently, single-cell RNA sequencing (scRNA-seq) has served as an effective method for identifying novel subpopulations of cells and revealing the differences of gene expression masked by bulk analysis in pooled cells, particularly for uncovering the heterogeneity of the immune system (13–18). Although studies have reported the dynamics and heterogeneity of individual immune cell subsets in acute or chronic infections as determined by scRNA-seq (19–22), the landscape of the entire lymphoid tissue immune cells has not yet been reported. Herein, utilizing scRNA-seq and flow cytometric analyses, we analyzed and compared the dynamics and heterogeneity of immune cells over time during acute and chronic infections on days 0, 4, 8 and 30 post-infection. Moreover, we revealed the difference in TCR and BCR usage during acute and chronic infections using scTCR-seq and scBCR-seq. This study provides new insights into the longitudinal maps of immune cells during acute and chronic viral infections and clarifies the transcriptional profiles and TCR/BCR repertoires of these cells at different times after infection.

## Materials and methods

### Mice and LCMV infection

The C57BL/6 mice, 6-8 wk old, were obtained from the Guangdong Medical Laboratory Animal Center (Guangdong, China) and housed under SPF conditions in the animal center of Shenzhen University. All mice were housed in a temperature-controlled room under a 12 h light/12 h dark cycle and pathogen-free conditions. All mouse experimental procedures were approved by the Animal Ethical and Welfare Committee of Shenzhen University (IACUC-202300026). The mice were injected intraperitoneally with 2×10^5^ PFUs (plaque-forming units) LCMV Armstrong to induce acute infection and injected intravenously with 2×10^6^ PFUs LCMV clone 13 to induce chronic infection, respectively.

### Viral titer quantification

The LCMV viral loads were quantified by Real-time quantitative RT-PCR (qRT-PCR). Total RNA was extracted from livers and lymph nodes using the Trizol reagent (Invitrogen, 15596018CN) and cDNA was synthesized by reverse transcription (TakaRa, RR047A) according to the manufacturer’s instructions. To quantify the LCMV viral load in livers and lymph nodes, LCMV-glycoprotein (GP) was measured by qRT-PCR (SYBR Green PCR Master Mix, Applied Biosystems, A25742), and the serial dilution of plasmid with LCMV-GP was used for standard curves. Primers for LCMV-GP: forward 5′-CAGGGGTGGAGAATCCAGGT-3′; reverse 5′-ATTTCGCAACTGCTGTGTTCC-3′.

### Lymph node dissociation and cell preparation

Single-cell suspensions were prepared as described (23) but with some modifications. Briefly, the fresh lymph nodes were washed using Hanks Balanced Salt Solution (HBSS) 3 times and digested using 2 ml sCelLiveTM Tissue Dissociation Solution (Singleron) by Singleron PythoN™ Automated Tissue Dissociation System (Singleron) for 15 mins at 37°C. The sample was then centrifuged for 5 minutes with 500 × g and suspended gently with PBS (HyClone). The cell suspension was stained with 7-AAD (BD Biosciences, 559925) and the 7-AAD^-^ viable CD45^+^ cells were sorted using FACS Aria II cell sorting system (BD Biosciences). Finally, the sorted cells were stained with trypan blue (Sigma, USA), and cell vitality was evaluated through a microscope.

### Library preparation and scRNA-seq

Single-cell suspensions (1×10^5^cells/ml) with PBS were loaded into microfluidic devices using the Singleron Matrix^®^ Single Cell Processing System (Singleron). Then, the scRNA-seq libraries were constructed referring to the protocol of the GEXSCOPE^®^ Single Cell RNA Library Kits (Singleron) (24). Individual libraries were diluted to 4 nM and pooled for sequencing. Finally, pools were sequenced on Illumina Nova6000 using 150 bp paired-end reads. scRNA-seq quantifications and statistical analysis were performed as previously described (23).

### Functional enrichment analysis of GO and KEGG

For GO and KEGG analysis, we utilized “enrichGO” and “enrichKEGG” functions from clusterProfiler (v4.10.0, R package) with org.Mm.eg.db (v3.18.0, R package), and we used “dotplot” function with 20 for “showCategory” parameter in enrichplot for the enrichment of Biological process (BP) (24, 25).

### Gene set enrichment analysis

For gene set enrichment analysis (GSEA), we referred to previously published methods (26). Briefly, the sample and group information was added to the data, and then use the “slot” parameter as the data to obtain the gene expression matrix through the “GetAssayData” function in Seurat.

### Flow cytometry analysis

Single-cell suspensions were incubated in FACS buffer containing Fc block reagent (BD Biosciences) and Ghost Dye violet 510 (1:100; Tonbo Biosciences), and then labeled with monoclonal antibody for 30 min at 4°C. The antibodies are diluted according to the manual unless otherwise indicated. Anti-CD8a (clone 53-6.7, BV605), anti-CD11b (clone M1/70, PE), anti-CD11c (clone N418, APC), anti-CD4 (clone RM4-5, BV605), anti-TCRβ (clone H57-597, PC7) and CD138(clone 281-2, PE) were purchased from Biolegend. Anti-NK1.1 (clone PK136, Percp), anti-CD19 (clone 1D3, APC), anti-Foxp3 (clone FJK-16s, PE), anti-CD25 (clone PC61.5, APC), anti-CD45(clone 30-F11, FITC/eFluor450), PD-1 (clone J43, APC), anti-B220 (clone RA3-6B2, APC-eFluor780), anti-SiglecH (clone eBio440c, BV421), anti-IFN-γ (clone XMG1.2, PE), anti-GzmA (clone GzA-3G8.5, PE) and anti-GzmB (clone QA16A02, BV421) were purchased from ThermoFisher Scientific. Anti-TIM-3 (clone RMT3-23) was purchased from Tonbo Bioscience. Anti-IgA (clone C10-3, FITC) was purchased from BD Biosciences.

To detect intracellular cytokine, the cells were stimulated by PMA (50 ng/ml, Sigma-Aldrich, P1585) and Ionomycin (1 μg/ml, Sigma-Aldrich, I0634) for 4h at 37°C with the protein transport inhibitor (Monensin: 1:1000, BD Biosciences, 554724 and Brefeldin A: 1:1000, BD Biosciences, 555029), and stained with Ghost Dye violet 510 before surface markers were stained. Then the cells were fixed and permeabilized using a BD Cytofix/Cytoperm kit (BD Pharmingen), and stained with specific intracellular cytokine antibodies. The flow cytometric data was acquired by Beckman Coulter CytoFlex S (Beckman Coulter, USA), and the data analysis was performed using FlowJo 10.8.1.

### qRT-PCR analysis

Real-time quantitative RT-PCR (qRT-PCR) analysis was performed using QuantStudio 3 PCR machine (Thermo Fisher Scientific, USA). Total RNA was extracted from the cell using the Trizol reagent (Invitrogen, USA). RNA was subjected to cDNA synthesis with a reverse transcription kit under the manufacturer’s protocol. qRT-PCR was performed using SYBR Green PCR Master Mix (Applied Biosystems, USA), and amplification primer sequences are listed in **Supplementary Table S1**.

### ELISA analysis

Serum LCMV-specific antibodies were measured by ELISA (27). Briefly, ELISA plates were coated with LCMV-infected BHK-21 cell lysate and blocked with blocking solution. Then 60µL serum from each mouse was diluted in 240 µL blocking solution (1:5), mixed and evenly distributed into three wells. Incubate for 90 min at room temperature. Then, wash plates three times with PBST. Dispense 100 µL goat anti-mouse IgA-HRP (ab97235, 1:10000 dilution) or goat anti-mouse IgG-HRP (ab6789, 1:100000 dilution) in blocking solution to each well. After that, incubate for 90 min at room temperature. After washing, 100µL TMB color-developing solution (Beyotime, P0210-100ml) was added. Incubate at room temperature for 8 minutes. Finally, 100µL TMB color-stop solution (Beyotime, P0215) was added to each well and read the optical density (O.D.) at 450 nm wavelength within 30 min immediately.

### scTCR and scBCR sequencing using BD rhapsody

scTCR and scBCR sequencing were performed as previously described but with some alterations (28). Briefly, the single-cell suspensions were incubated with Ghost Dye™ Violet 510 for 15 min at room temperature, CD4^+^ T cells, CD8^+^ T cells and CD19^+^ B cells were sorted by FACS. Then, the cells were incubated with Fc block reagent (BD Pharmingen) and Sample Tag antibodies at room temperature for 20 min. The sorted three subsets from the same sample were sequentially labeled using BD Mouse Immune Single-Cell Multiplexing Kit conjugated to an Anti-Mouse CD45 antibody (Clone 30-F11) and BD AbSeq Ab-Oligos reagents according to the manufacturer’s protocol (BD Pharmingen).

The cDNA was prepared by performing random priming and extension (RPE) on BD Rhapsody Cell Capture Beads and then cDNA underwent targeted amplification using sample Tag PCR Primer, BD AbSeq Primer (11 cycles), TCR and BCR (15 cycles) via PCR. The products were purified using Agencourt AMPure XP Beads (Beckman Coulter). Final libraries sequenced by Illumina NovaSeq 6000 on a 150 bp paired-end run.

### scTCR-seq and scBCR-seq analysis

scTCR-seq and scBCR-seq analysis were performed according to previously published methods with partial modifications (29). Briefly, the Cell Ranger VDJ pipeline was applied to assemble TCR or BCR sequences and then identify CDR3 and TCR or BCR genes. The TCR/BCR VDJ results were added to the meta.data of the Seurat object by using the AddMetaData function. The highest UMIs chain was retained if multiple α or β chains in a cell were detected (30). Cells with a pair of TCR α/β chains or BCR heavy/light chains that appeared in at least three cells were defined as expanded clonal cells.

### Statistical analyses

Statistical analyses in this study were performed using GraphPad Prism 8.0(GraphPad Software, Inc., San Diego, CA). Data were analyzed using ordinary one-way analysis of variance (ANOVA) when comparing multiple groups or an unpaired Student’s t-test when comparing two groups as indicated. *P values < 0.05, **P values < 0.01, ***P values < 0.001.

## Results

### Single-cell analysis of dynamic changes in immune cells over the course of acute and chronic infections

**Figure 1:**
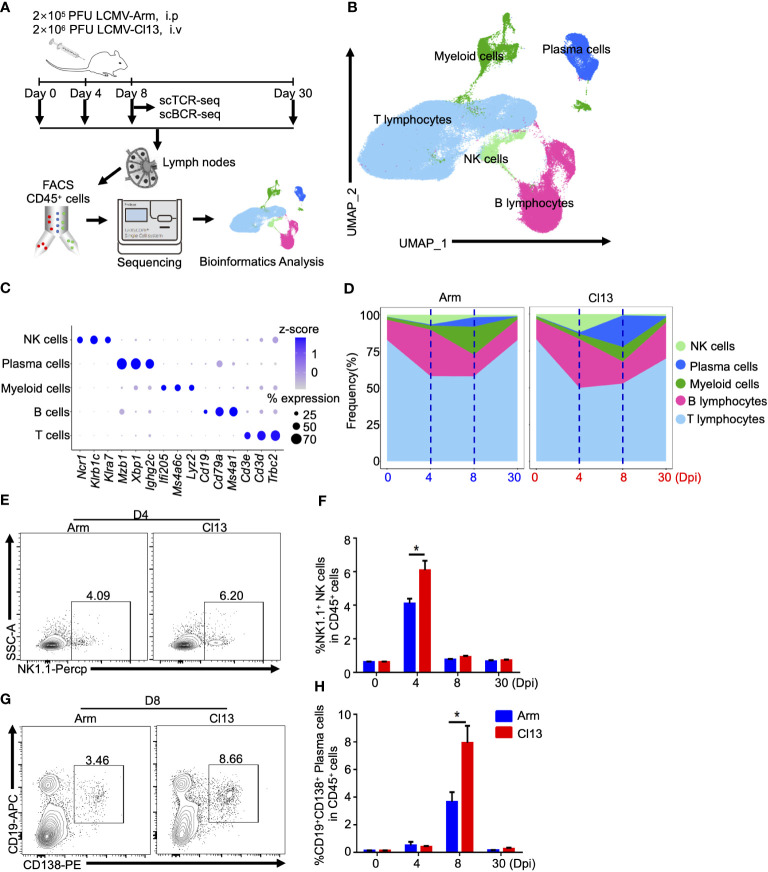
The dynamics of immune cells with scRNAseq. **(A)** Schematic of experimental design. **(B)** Uniform manifold approximation and projection (UMAP) visualization of the cell-type composition of the assayed samples by single cell transcriptome profiles. Distinct cell types are depicted with different colors. **(C)** Dot plot of marker genes for distinct cell types. Color scale indicates the mean of normalized expression of marker genes in each cell type, and dot size is proportional to the percentage of cells within each cell cluster expressing the marker genes. Cell cluster IDs on the left correspond to those in **(B)**. **(D)** Proportion of each defined cell type across groups. Color scale and size scale are the same as those in **(B)**. **(E–H)** Flow cytometry of NK1.1^+^ NK cells and CD19^+^ CD138^+^ plasma cells in lymph nodes of mice infected with virus for days 0, 4, 8 and 30. **(F)** Plots are gated on CD45^+^ cells. **(E)** The percentages of the NK1.1^+^ NK cells are shown in representative Dot Plot and **(F)** cumulative data on the frequency are summarized in bar graphs (n = 6 from 2 independent experiments). **(G)** Plots are gated on CD45^+^ cells. The percentages of the CD19^+^CD138^+^ plasma cells are shown in representative Dot Plot and **(H)** cumulative data on the frequency are summarized in bar graphs (n = 6 from 2 independent experiments). *, P < 0.05 (Student’s t-test).

To assess the dynamics of immune cells at different stages of acute and chronic infections, CD45^+^ immune cells from the lymph nodes of mice with acute LCMV Armstrong (Arm) infection or chronic LCMV clone 13 (Cl13) infection at 0 (Naive), 4, 8 and 30 days post-infection (dpi) were isolated by fluorescence-activated cell sorting (FACS) and subjected to scRNA-seq or TCR/BCR sequencing (**Figure 1A**). After excluding low-quality cells, potential doublets and dead cells, we obtained a total of 158034 cells from Day 0 (25,173 cells) across 2 biological replicates, Day 4 (20,628 (Arm) and 20,262 (Cl13) cells across two biological replicates, Day 8 (21,797 (Arm) and 24,932 (Cl13) cells across two biological replicates, and Day 30 (22,062 (Arm) and 23,180 (Cl13) cells across two biological replicates. Cluster analysis of these cells identified five distinct clusters with specific temporal progression characteristics when visualized on a uniform manifold approximation and projection (UMAP) plot (**Figure 1B**). Clusters corresponding to natural killer (NK) cells, plasma cells, myeloid cells, B lymphocytes and T lymphocytes were identified based on known markers (31–33). These markers included *Ncr1*, *Klrb1c* (NK1.1), and *Klra7* for NK cells; *Mzb1*, *Xbp1*, and *Ighg2c* for plasma cells; *Ifi205*, *Ms4a6c*, and *Lyz2* for myeloid cells; *Cd19*, *Cd79a*, and *Ms4a1* for B lymphocytes; and *Cd3g*, *Cd3g*, and *Trbc2* for T lymphocytes (**Figure 1C**).

Dynamic changes in all five immune cell subsets were described over the course of acute and chronic viral infections (**Figures 1D**, **S1B**). Notably, NK cells and B cells in both acutely and chronically infected mice were activated and expanded at the early stage (4 dpi), the peak time point for the proportions of NK and B cells during viral infection (**Figures 1D**, **S1B**). In contrast, myeloid cells and plasma cells were subsequently expanded at the acute effector stage (8 dpi) (**Supplementary Figures S1B, D**).

Among innate immune cells, NK cells exhibited a higher frequency during chronic infection (12.3%) than acute infection (6.6%) on 4 dpi. After, the proportion of NK cells decreased comparably in the two groups (**Figures 1D**, **S1B**). Flow cytometric analysis also validated the change in the proportion of NK cells during viral infection (**Figures 1E, F**, **S1C**). Furthermore, Gene Ontology (GO) enrichment analysis and gene set enrichment analysis (GSEA) showed that NK cells were functionally impaired in response to type I interferon or to chronic viral infection (**Supplementary Figures S1G–I**). In addition, the proportion of myeloid cells peaked at the acute effector stage (8 dpi), with a much higher frequency in acute infection (19.0%) than chronic infection (9.8%), and subsequently decreased (30 dpi) comparably in the two groups (**Figures 1D**, **S1B**).

Regarding adaptive immune cells, interestingly, the proportion of B cells peaked at the early stage (4 dpi) (**Figure 1D**) and then declined (8 dpi) during both acute infections (0 dpi, 13.7%; 4 dpi, 31.7%; 8 dpi, 14.7%, respectively) and chronic infections (0 dpi, 13.7%; 4 dpi, 33.1%; 8 dpi, 14.9%, respectively); this decrease was followed by a moderate rebound during chronic infection at the persistent infection stage (30 dpi,14.1% in acute infection and 24.4% in chronic infection) (**Figures 1D**, **S1B, C, E**). Subsequently, plasma cells differentially expanded at the acute effector stage (6.5%) (8 dpi) following the previous expansion of B cells, which also had a higher frequency during chronic infection (21.4%) (**Figures 1D, G, H**). Additionally, there was a slight decrease in the proportion of T lymphocytes during chronic infection, with the proportion of T cells decreasing soon after infection and rebounding on 30 dpi in both groups (**Figures 1D**, **S1B, F**).

### Cluster analysis of myeloid cells showed a diminished number but aberrant activation of pDCs during chronic infection

**Figure 2:**
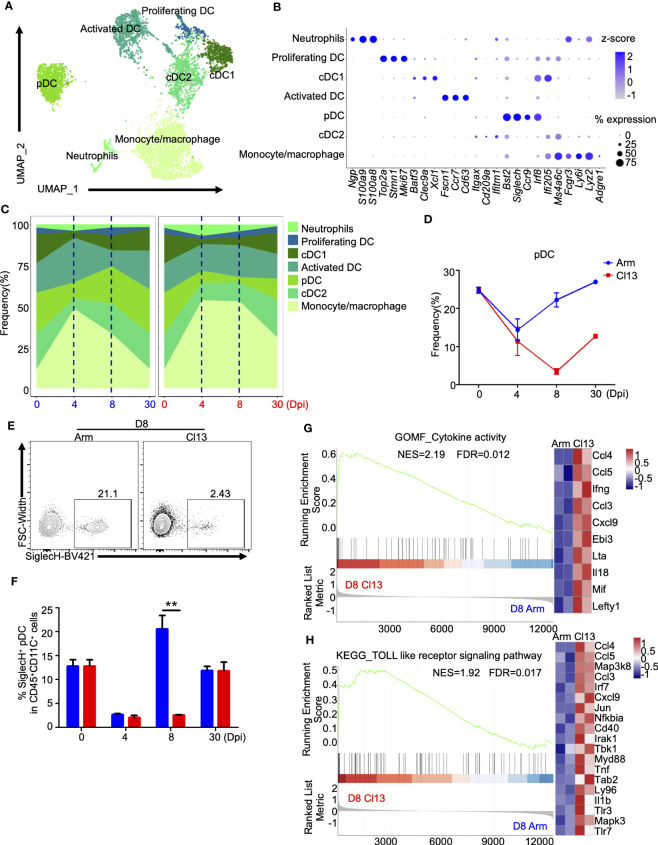
Myeloid clusters analysis showed that pDC plays an important role in the formation of chronic infection. **(A)** UMAP plot of all myeloid cells from days 0 and 4, 8, and 30 (**Figure 1B**) were extracted, reclustered, and re-embedded in new UMAP coordinates. Cells are colored by myeloid subtype as shown in legend on right. **(B)** Dot plot of marker genes for distinct cell types. Color scale indicates the mean of normalized expression of marker genes in each cell type, and dot size is proportional to the percentage of cells within each cell cluster expressing the marker genes. Cell cluster IDs on the left correspond to those in **(A)**. **(C)** Proportion of each defined cell subsets across groups. **(D)** Percentage of each identified pDC across groups during Arm (LCMV-Armstrong) and Cl13 (LCMV-Cl13) infections. **(E, F)** Flow cytometry of SiglecH^+^pDC cells in lymph nodes of mice infected with virus for 8 days. Plots are gated on CD45^+^CD11c^+^ cells. **(E)** The percentages of the SiglecH^+^pDC cells are shown in representative Dot Plot and **(F)** cumulative data on the frequency are summarized in bar graphs (n = 6 from 2 independent experiments). **, P < 0.01 (Student’s t-test). **(J, H)** GSEA showing enriched expression of genes in the pDCs derived from Cl13 infection and Arm infection group for 8 dpi, with the enriched genes displayed in a heatmap. NES, normalized ES; FDR, false discovery rate; p, normalized p value.

To further understand the heterogeneity of myeloid cells, we conducted a detailed clustering analysis and identified 7 different subtypes (**Figure 2A**). Clusters corresponding to monocytes/macrophages, cDC2s, pDCs, activated DCs, cDC1s, proliferating DCs and neutrophils were identified based on previously published markers (**Figure 2A**) (31, 33). These included *Lyz2*, *Ly6i*, *Fcgr3*, and *Ms4a6c* for monocytes/macrophages; *Itgax* (CD11c) and *Cd209a* for cDC2s; *Ccr9*, *Siglech*, and *Bst2* for pDCs; *Cd63*, *Ccr7*, and *Fscn1* for activated DCs; *Xcl1*, *Clec9a*, and *Batf3* for cDC1s; *Mki67*, *Stmn1*, and *Top2a* for proliferating DCs; and *S100a8*, *S100a9*, and *Ngp* for neutrophils (**Figure 2B**).

In contrast to DCs, neutrophils and monocytes/macrophages were preferentially expanded at the early stage during both acute and chronic infections, and their proportions peaked and then declined during acute infection (**Supplemenatry Figure S2A**). However, higher proportions of neutrophils and monocytes/macrophages were maintained during chronic infection until the persistent infection stage (30 dpi) (**Figures 2C**, **S2A**). Unlike those of neutrophils and monocytes/macrophages, the proportion of cDCs first decreased and then increased during both acute and chronic infection (**Figures 2C**, **S2A**). At the acute effector stage (8 dpi) (27.5%), the proportion of cDCs (cCD1 and cCD2) was lower during chronic infection (16.4%) (**Figures 2C**, **S2A**). Additionally, the proportion of activated DCs decreased consistently during the early stage of both acute and chronic infections, and this decrease was followed by different dynamic changes at the acute phase (8 dpi) and persistent stage (30 dpi) (**Figures 2C**, **S2A**).

Plasmacytoid dendritic cells (pDCs) exert an early and strong response to viral infection. The proportion of pDCs decreased significantly at the early stage during both acute and chronic infections (**Figures 2C, D**). Interestingly, the pDCs frequency in acute infection started to rebound at the acute phase (8 dpi) (0 dpi, 25.0%; 4 dpi, 9.8%; 8 dpi, 22.5%, respectively) to the baseline frequency before infection, but this phenomenon did not occur during chronic infection (0 dpi, 25.0%; 4 dpi, 7.3%; 8 dpi, 3.3%, respectively), despite the delayed rebound after the acute effector stage (8 dpi) (**Figures 2C, D**), which was also validated by flow cytometric analysis (**Figures 2E, F**). GO (**Supplementary Figure S2B**) and KEGG (**Supplementary Figure S2C**) enrichment analyses of differentially expressed genes (DEGs) indicated the differential immune responses of pDCs between acute and chronic viral infections. Furthermore, GSEA of the differentially expressed genes in pDCs (8 dpi) showed that pDCs during chronic infection exhibited significantly higher enrichment of cytokine activity (NES=2.19, FDR=0.012) and the Toll-like receptor signaling pathway (NES=1.2, FDR=0.01), exhibiting upregulation of *Lefty1*, *Mif*, *Il18*, *Lta*, *Ccl3*, *Ccl4* and *Ccl5* in the cytokine-mediated signaling pathway and upregulation of *Tlr7*, *Tlr3*, *Myd88*, *Nfkbia*, *Jun* and *Irf7* in the TLR signaling pathway (**Figures 2G, H**). In addition, transcriptional profiling showed that pDCs expressed higher levels of *Nkg7*, *Map3k8*, *Cd69*, *Ifitm3*, *Ifi44*, *Ifi207*, *Lgals3bp*, *Isg15*, *Gm30211*, *Ifi27l2a*, *Ccl5*, *Cd4*, *Ifit2*, *Cd8b1*, *Ifi209* and *Ccl4* and lower levels of *Mir6236*, *Cd7*, *Sell*, *Itgax* and *Cd180* during chronic infection (**Supplementary Figures S2D-H**).

### B-cell analysis showed that chronic viral infection is more inclined to activate the mucosal immune response than acute viral infection

**Figure 3:**
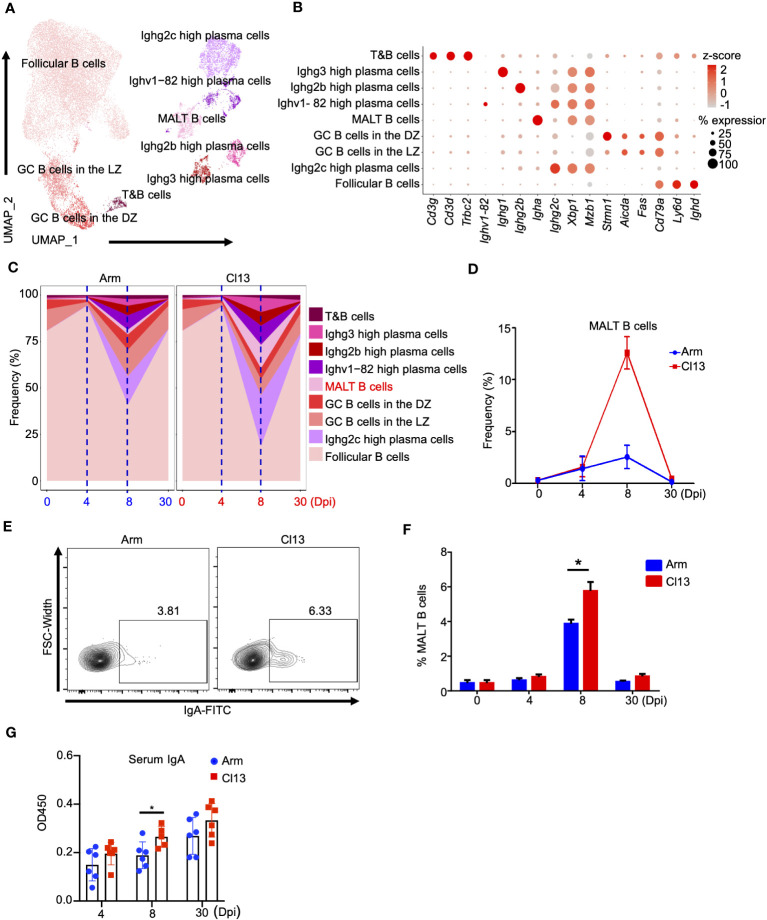
B cells analysis showed that chronic viral infection is more likely to activate mucosal immune response than acute viral infection. **(A)** UMAP plot of all B cells from days 0 and 4, 8, and 30 (**Figure 1B**) were extracted, reclustered, and re-embedded in new UMAP coordinates. Cells are colored by B cells subtype as shown in legend on right. **(B)** Dot plot of marker genes for distinct cell types. Color scale indicates the mean of normalized expression of marker genes in each cell type, and dot size is proportional to the percentage of cells within each cell cluster expressing the marker genes. Cell cluster IDs on the left correspond to those in **(A)**. **(C)** Proportion of each defined cell type across groups. **(D)** Percentage of each identified cell subtype MALT B cells across groups during Arm (LCMV-Armstrong) and Cl13 (LCMV-Cl13) infections. **(E)** Flow cytometry of CD19^+^ IgA^+^ MALT B cells in lymph nodes of mice infected with virus for days 0, 4, 8 and 30. Plots are gated on CD45^+^CD19^+^ B cells. The percentages of the IgA ^+^ MALT B cells are shown in representative Dot Plot and **(F)** cumulative data on the frequency are summarized in bar graphs (n = 6 from 2 independent experiments). **(G)** The secretion of IgA is analyzed by ELISA. *, P < 0.05(Student’s t-test).

To better understand B lymphocyte heterogeneity during chronic and acute infections, we conducted further cluster analysis of B lymphocytes and determined 9 different subtypes (**Figure 3A**). Clusters corresponding to follicular B cells, Ighg2c-high plasma cells, GC B cells in the LZ, GC B cells in the DZ, MALT B, Ighv1-82-high plasma, Ighg2b-high plasma and Ighg3-high plasma cells were identified based on known markers (**Figures 3A, B**) (34). These included *Ighd*, *Ly6d*, and *Cd79a* for follicular B cells; *Mzb1*, *Xbp1*, and *Ighg2c* for Ighg2c-high plasma cells; *Fas* and *Aicda* for GC B cells in the LZ; *Stmn1* for GC B cells in the DZ; *Igha* for MALT B cells; *Ighv1-82* and *Ighg2c* for Ighv1-82-high plasma cells; *Ighg2b* for Ighg2b-high plasma cells; and *Ighg3* for Ighg3-high plasma cells (**Figure 3B**).

As mentioned above, the proportion of B cells peaked at the early stage (4 dpi) and then declined during both acute and chronic infections (**Figure 1D**). Subsequently, plasma cells expanded at the acute effector stage (8 dpi), especially during chronic infection (**Figures 1D, H**, **S1B**). In detail, follicular B cells significantly declined on day 8 of infection and rebounded to a proportion comparable to that before infection on 30 dpi both acute infections (0 dpi, 80.7%; 4 dpi, 94.3%; 8 dpi, 40.9%; 30 dpi, 81.5%, respectively) and chronic infections (0 dpi, 80.7%; 4 dpi, 93.5%; 8 dpi, 18.5%; 30 dpi, 77.1%, respectively) (**Figures 3C**, **S3A**). The proportions of GC B cells in the dark zone (DZ) and GC B cells in the light zone (LZ) were lowest on 4 dpi and then rebounded to the preinfection proportions (**Figures 3B**, **S3A**), with the proportion of GC B cells lower during chronic infection on 8 dpi (**Figures 3B**, **S3A**). Interestingly, the proportions of plasma cells, especially Ighg3-high and Ighg2c plasma cells, were significantly increased during chronic infection on 8 dpi (**Figures 3B**, **S3A**) but exhibited no significant difference on 4 and 30 dpi (**Figures 3B**, **S3A**).

Of note, on 8 dpi, IgA^+^ plasma cells (MALT B cells) in chronic infection (12.8%) were significantly higher than those in acute infection (2.4%) (**Figures 3C, D**), and this finding was also validated by a flow cytometric analysis (**Figures 3E, F**). Correspondingly, the level of secreted virus-specific IgA in serum was also higher during chronic infection than acute infection on 8 dpi (**Figure 3G**). Still, the differences at other time points were insignificant (**Figure 3G**). Surprisingly, regardless of the time since infection, the difference in the level of secreted virus-specific IgG in serum during chronic infection and acute infection was not obvious (**Supplementary Figure S3B**). This indicated that chronic viral infection is more likely to activate the mucosal immune response than acute viral infection. In addition, we also identified a minor population expressing both B and T cell lineage markers (**Figure 3B**), namely, T&B cells (35).

### The clonal diversity of B cells revealed by scBCR-seq during acute and chronic infections

**Figure 4:**
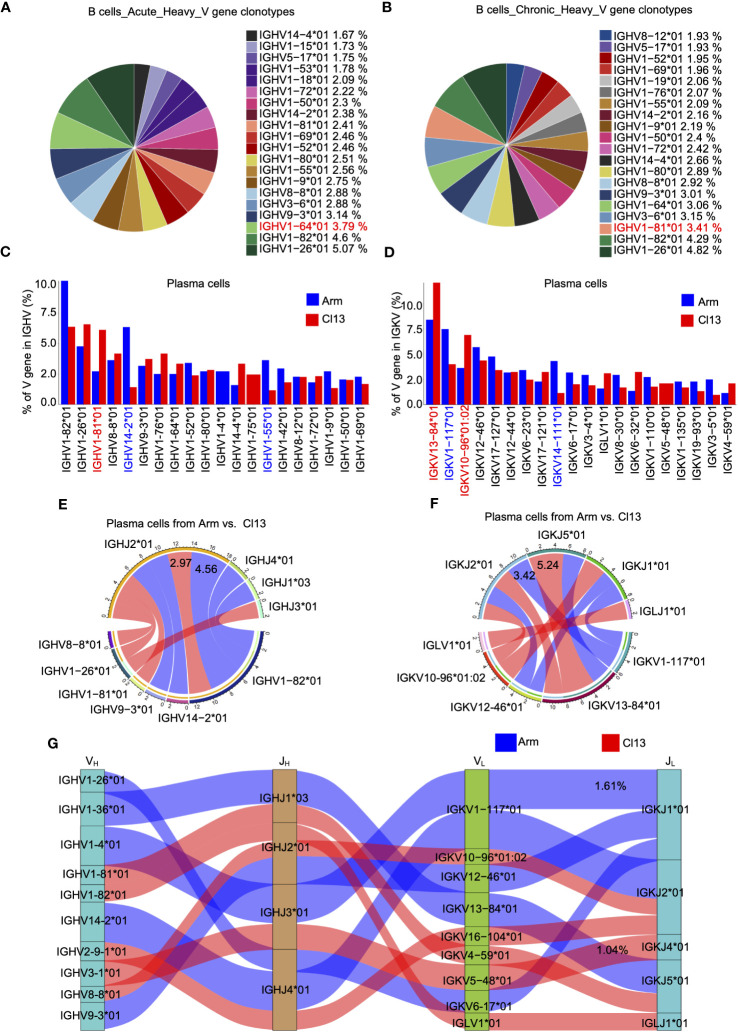
The clonal diversity of B cell revealed by scBCR-seq during acute and chronic infection. Pie chart showing the usage top 20 of BCR heavy chain V genes in plasma cells from the acute **(A)** and chronic **(B)** groups, respectively.Bar plot showing usage of top 20 BCR heavy **(C)** and light **(D)** V genes in plasma cells from the acute and chronic groups. Circos plots show the differential heavy **(E)** and light **(F)** VJ pairs in plasma cells from the acute and chronic groups. Blue links represent acute groups specific VJ pairs, and red links represent chronic groups specific VJ pairs. **(G)** Sankey diagram shows significant different frequency of heavy-light VJ pairs in plasma cells from the acute and chronic groups. Blue links represent acute groups specific pairs, and red links represent chronic groups specific pairs.

The diversity of antibody recognition is determined by V(D)J rearrangement and somatic hypermutation (SHM), and B-cell receptor (BCR) heavy chains with the rearrangement of the V, D and J genes showed greater diversity than BCR light chains (36). Herein, we first analyzed the usage of BCR heavy chain V genes among total B cells (8 dpi) during acute and chronic infections. The results showed that acute and chronic infections exhibited uniquely preferential usage of the V gene among the top 5 V genes, namely, IGHV1−64*01 (3.79%) in acute infection (**Figure 4A**) and IGHV1−81*01 (3.41%) in chronic infection (**Figure 4B**). Furthermore, we evaluated the usage of BCR heavy and light chain V genes (IGHV and IGKV, respectively) in plasma cells. Our data showed that there were high levels of IGHV1-82*01 and IGHV14-2*01 expression in plasma cells during acute infection but a high level of IGHV1-81*01 expression during chronic infection (**Figure 4C**). Moreover, there were high levels of light chain IGKV1−117*01 and IGKV14−111*01 expression in plasma cells during acute infection but high levels of IGKV13−84*01 and IGKV10−96*01:02 expression during chronic infection (**Figure 4D**). Among the heavy chain VJ pairs, the IGHV1-82*01_IGHJ2*01 pair (4.56%) had the highest percentage in the Arm group, but the IGHV1-82*01_IGHJ2*01 pair (2.97%) had the highest percentage in the Cl13 group (**Figure 4E**). Among the light chain VJ pairs, the IGKV1-117*01_IGKJ2*01 pair (3.42%) had the highest percentage in the Arm group, but the IGKV13-84*01_IGKJ5*01 pair (5.24%) had the highest percentage in the Cl13 group (**Figure 4F**). Among the heavy-light chain VJ pairing profiles, the most common VJ pairing profile in expanded clones was IGHV1-4*01_IGHJ3*01_IGKV1_117*01_IGKJ1*01 (1.61%), which was the most dominant clonotype in plasma cells during acute infection, but the most common VJ pairing profile during chronic infection was IGHV3-1*01_IGHJ3*01_IGKV5-48*01_IGKJ4*01 (1.04%) (**Figure 4G**).

### T-cell cluster analysis indicated that Gzma-high effector-like CD8^+^ T cells have different genetic features and clonal diversity during acute and chronic infections

**Figure 5:**
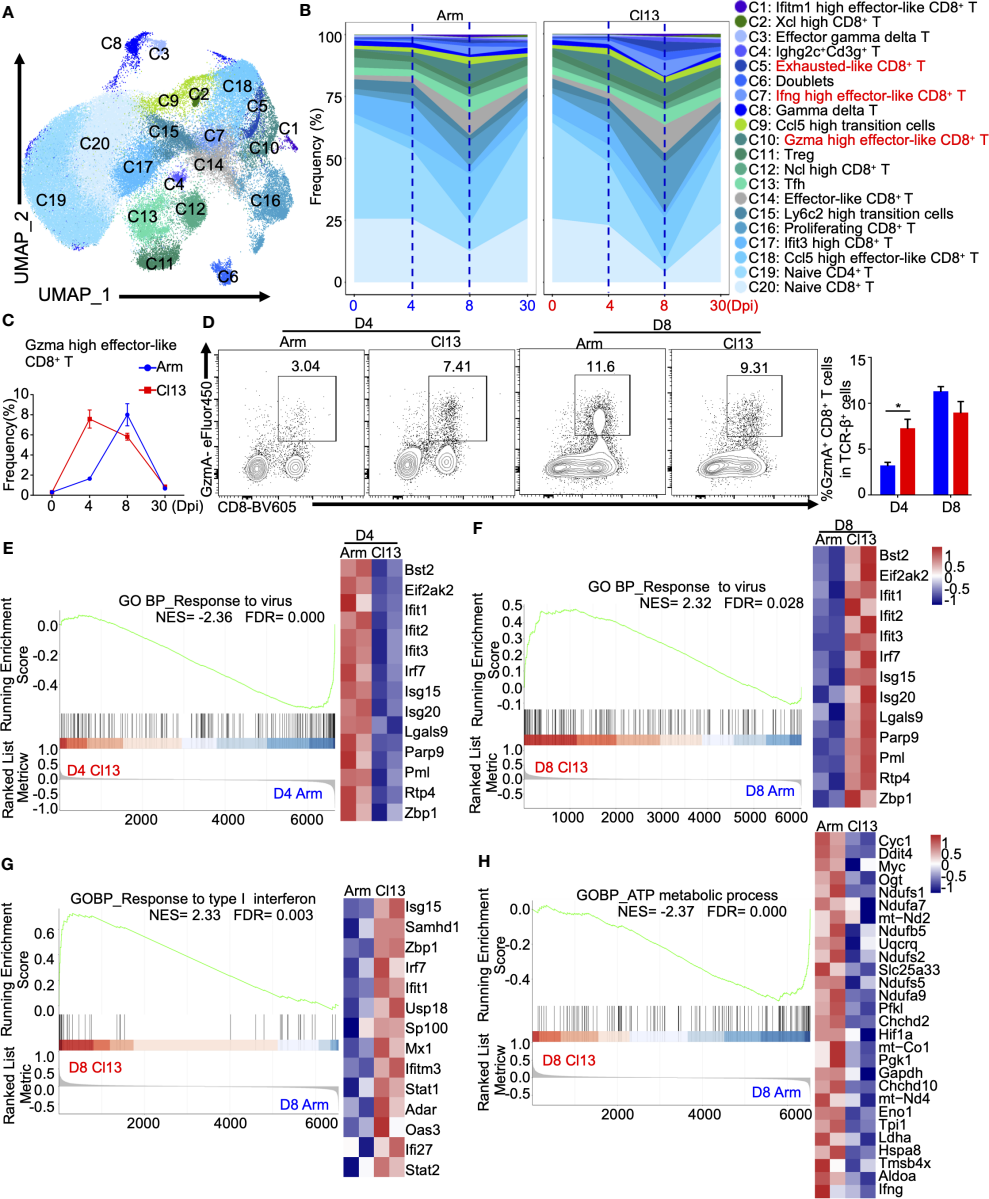
The dynamics and heterogeneity of T cell clusters during acute and chronic virus infections. **(A)** UMAP plot of all T cells from days 0, 4, 8, and 30 during Arm (LCMV-Armstrong) and Cl13 (LCMV-Cl13) infections. (**Figure 1B**) were extracted, reclustered, and re-embedded in new UMAP coordinates. Cells are colored by T cells subtype as shown in legend on right. **(B)** Proportion of each defined cell type across groups. **(C)** Percentage of each identified cell subtype Gzma high effector-like CD8^+^ T across groups during Arm and Cl13 infections. **(D)** Flow cytometry of Gzma high effector-like CD8^+^ T cells in lymph nodes of mice infected with virus on the 4 and 8 days. **(E)** Percentage of each identified cell subtype Ifng high effector-like CD8^+^ T across groups during Arm and Cl13 infections. **(F)** Flow cytometry of Ifng high effector-like CD8^+^ T cells in lymph nodes of mice infected with virus on the 4 and 8 days. GSEA showing enriched expression of genes in the Gzma high effector-like CD8^+^ T derived from Cl13 infection and Arm infection group for 4 **(G)** and 8 dpi **(H)**, with the enriched genes displayed in a heatmap. GSEA showing enriched expression of genes in the Gzma high effector-like CD8^+^ T derived from Cl13 infection and Arm infection group for 4 **(G)** and 8 dpi **(H)**, with the enriched genes displayed in a heatmap. **(I, J)** GSEA showing enriched expression of genes in the Ifng high effector-like CD8^+^ T derived from Cl13 infection and Arm infection group for 8 dpi, with the enriched genes displayed in a heatmap. NES, normalized ES; FDR, false discovery rate; p, normalized p value.

Although the dynamics of T cell frequencies are similar between acute and chronic infections, the subpopulations within T lymphocytes could differ. To investigate T lymphocyte heterogeneity, we performed cluster analysis of T lymphocytes and identified 20 distinct subtypes (**Figures 5A, B**). Clusters corresponding to naive CD8^+^ T (C20), naive CD4^+^ T (C19), Ccl5-high effector-like CD8^+^ T(C18), Ifit3-high CD8^+^ T (C17), proliferating CD8^+^ T(C16), Ly6c2-high transition (C15), effector-like CD8^+^ T (C14), Tfh (C13), Ncl-high CD8^+^ T (C12), Treg (C11), Gzma-high effector-like CD8^+^ T (C10), Ccl5-high transition (C9), gamma delta T (C8), Ifng-high effector-like CD8^+^ T (C7), doublet (C6), exhausted-like CD8^+^ T (C5), Ighg2c^+^Cd3g^+^ T (C4), effector gamma delta T (C3), Xcl-high CD8^+^ T (C2) and Ifitm1-high effector-like CD8^+^ T cells (C1) were identified based on known markers (21, 37)(**Figures 5A, B**, **S4A, B**). Herein, CD8^+^ T cells included naive CD8^+^ T cells, marked by *Cd3g*, *Cd8b1*, *Sell*, and *Il7r*; Ccl5-high effector-like CD8^+^ T cells, marked by *Ccl5*; Ifit3-high CD8^+^ T cells, marked by *Ifit3*; proliferating CD8^+^ T cells, marked by *Mki67*; Ly6c2-high transition cells, marked by *Ly6c2*; effector-like CD8^+^ T cells, marked by *Gzmb*; Ncl-high CD8^+^ T cells, marked by *Ncl*; Gzma-high effector-like CD8^+^ T cells, marked by Gzma; Ccl5-high transition cells, marked by *Ccl5* and *Sell*; gamma delta T cells, marked by *Trdc*, *Tcrg−C2*, and *Tcrg−C4*; Ifng-high effector-like CD8^+^ T cells, marked by *Ifng*; exhausted-like CD8^+^ T cells, marked by *Pdcd1*, *Ccl3*, and *Ccl4*; effector gamma delta T cells, marked by *Tcrg-c1*, *Tmem176b*, and *Tmem176a*; Xcl-high CD8^+^ T cells, marked by *Xcl*; and Ifitm1-high effector-like CD8^+^ T cells, marked by *Ifitm1*.

Among the defined CD8^+^ T-cell subsets, Gzma-high effector-like CD8^+^ T cells expressed higher levels of *Gzma*, *Lgals1*, *S100a6*, *Id2*, *Gzmk*, *Anxa2*, *Crip1*, *Klrg1* and *Cd48* during both acute and chronic infection (**Supplementary Figures S4C, D**), in which they are commonly called short-lived effector cells (SLECs) (38), but higher levels of *Havcr2* and *Pdcd1* during *chronic infection* (**Supplementary Figure S4D**). Intriguingly, the production of Gzma-high effector-like CD8^+^ T cells was induced earlier and faster at early stage during chronic infection (**Figures 5B, C**). On 4 dpi, the proportion of Gzma-high effector-like CD8^+^ T cells in chronic infection (7.3%) was significantly higher than that in acute infection (1.6%) (**Figures 5C, D**), in which it peaked on 8 dpi (**Figures 5C, D**). Furthermore, transcriptional analysis showed that Gzma-high effector-like CD8^+^ T cells expressed high levels of the effector T-cell-associated genes *Ccl5*, *Klrg1*, *Jak1*, *Lck*, *Cx3cr1*, *Id2*, *S100a10*, *Klrc1* and *Slamf7* during acute infection but high levels of T-cell exhaustion-associated genes *Ifi27i2a*, *Isg15*, *Lgals3bp*, *Bst2* and *Pdcd1* during chronic infection (**Supplementary Figure S4E**). Interestingly, the gene expression profile related to responses to virus of Gzma-high effector CD8^+^ T cells was temporally reversed throughout viral infection, and the GO (**Supplementary Figure S4F**) and GSEA (**Figure 5E**) analyses showed that Gzma-high effector-like CD8^+^ T cells had significantly lower gene enrichment in response to viral infection on 4 dpi (**Figure 5E**) but higher enrichment on 8 dpi during chronic infection than during acute infection (**Figures 5F**, **S4F**).

In addition, Ifng^high^ effector-like CD8^+^ T cells, which expressed higher levels of *Ifng*, *Gzmb*, *AW112010*, *Hspa5*, *Fcer1g*, *Plac8*, *Cxcr6*, *Gapdh*, *Havcr2* and *Prf1* (**Supplementary Figures S4B, G**), were greatly expanded on 8 dpi during both acute and chronic infections, although a slightly higher proportion was observed during chronic infection (**Supplementary Figures S4H, I**). GO biological process enrichment analysis and GSEA of differentially expressed genes in Ifng^high^ effector-like CD8^+^ T cells also showed differential regulation between acute and chronic infections (**Supplementary Figures S4J, K**), manifested as significantly higher enrichment of the response to interferon-gamma (**Figure 5G, H**; **Supplementary Figure S4L** upper panel) and lower enrichment of the terms regulation of ATP, metabolic process and T-cell mediated cytotoxicity during chronic infection.

Chronic viral infection can induce functional exhaustion in CD8^+^ T cells. Given that exhausted-like CD8^+^ T cells and Ifng-high effector-like CD8^+^ T cells express high levels of genes associated with exhaustion markers, such as *Pdcd1*, *Lag3* and *Havcr2* (TIM3), we analyzed the transcriptional features of exhausted-like CD8^+^ T cells and Ifng-high effector-like CD8^+^ T cells during both acute and chronic infection (**Supplementary Figure S4M**). The results showed that Ifng-high effector-like CD8^+^ T cells expressed higher levels of *Gzmb*, *Ly6e*, *Cxcr6*, *Id2*, *Ifngr1*, *Ccr2*, *Isg15* and *Cd47* than exhausted-like CD8^+^ T cells, which expressed higher levels of *Ccl4*, *Ccl3*, *Ccl5*, *Ifi27l2a*, *Nr4a2*, *Ifng*, *Tox* and *Bcl2a1b*, Pdcd1, lag3 and havcr2 (**Supplementary Figure S4M**).

### The clonal diversity of CD8^+^ T cells revealed by scTCR-seq during acute and chronic infections

**Figure 6:**
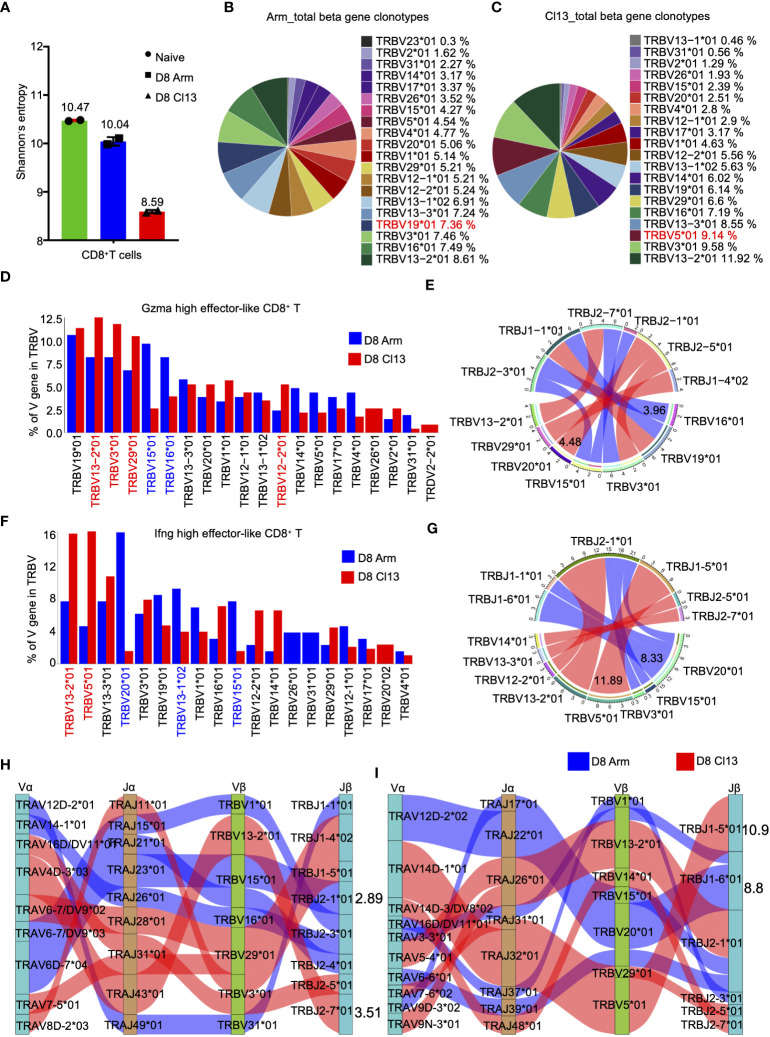
The clonal diversity of CD8^+^ T cell revealed by scTCR-seq during acute and chronic infection. **(A)** Analysis of the clonal expended TCR β chain. Bar graphs showing the clonal diversity of CD8^+^ T cells from Arm and Cl13 (8 dpi), which was calculated using Shannon’s entropy. Pie chart showing the usage top 20 of TCR heavy chain V genes in CD8^+^ T cells from the Arm **(B)** and Cl13 **(C)** groups, respectively. **(D)** The bar plots showing usage of some V genes from Gzma high effector-like CD8^+^ T cells TCR β chain of Arm group (blue)and Cl13 group (red) (8 dpi) (Top20). **(E)** Circos plots show the differential TCR β VJ pairs in Gzma high effector-like CD8^+^ T cells from the Arm and Cl13 groups. Blue links represent acute groups specific VJ pairs, and red links represent chronic groups specific VJ pairs. **(F)** The bar plots showing usage of some V genes from Ifng high effector-like CD8^+^ T cells TCR β chain of Arm group (blue)and Cl13 group (red) (8 dpi) (Top20). **(G)** Circos plots show the differential TCR β VJ pairs in Ifng high effector-like CD8^+^ T cells from the Arm and Cl13 groups. Blue links represent acute groups specific VJ pairs, and red links represent chronic groups specific VJ pairs. **(H)** Sankey diagram shows significant different frequency of α-β VJ pairs in Gzma high effector-like CD8^+^ T cells **(H)** and Ifng high effector-like CD8^+^ T cells **(I)** from the Arm and Cl13 groups. Blue links represent Arm groups specific pairs, and red links represent Cl13 groups specific pairs.

Gene rearrangement at TCR α/β loci generates highly diverse complementarity‐determining regions (CDRs), where the third CDR (CDR3) is the most hypervariable, thus generating a functional and highly diverse TCR repertoire (39, 40). Herein, we used the Shannon entropy index to calculate the diversity of the TCR repertoire (39, 40), which is positively correlated with the diversity of the CDR3 clone. The entropy indices of CD8^+^ T cells under naive conditions, acute and chronic infection were 10.47, 10.04, and 8.59, respectively (**Figure 6A**), indicating the reduced diversity of CD8^+^ T cells during chronic infection. In addition, among the top 5 TCR heavy chain V genes, differential usage of V genes was observed during acute and chronic infection, with higher usage of TRBV19*01 (7.36%) for acute infection (**Figure 6B**) and higher usage of TRBV5 * 01 (9.14%) for chronic infection, respectively. (**Figure 6C**).

The diversity of T-cell receptors is determined by the variable regions on the α chain and β chain, encoded by *TRAV* and *TRBV*, respectively. Herein, the expression of *TRBV* and *TRAV* genes in Gzma-high effector-like CD8^+^ T cells was analyzed, revealing a high proportion of TRBV15*01 and TRBV16*01 during acute infection but high proportions of TRBV13−2*01, TRBV3*01 and TRBV29*01 during chronic infection (**Figure 6D**). Among the β chain VJ pairs, the TRBV16*01_TRBJ2-3*01 pair (3.96%) had the highest percentage in the acute infection group, but the TRBV20*01_TRBJ2-5*01 pair (4.48%) had the highest percentage in the Cl13 group (**Figure 6E**). Our data also showed high levels of TRAV6D−7*04, TRAV14−1*01 and TRAV12D−2*02 expression in Gzma-high effector-like CD8^+^ T cells in the Arm group but high levels of TRAV13D−1*01, TRAV4D−3*03 and TRAV16*01 expression in the Cl13 group (**Supplementary Figure S5A**). Among the α chain VJ pairs, the TRAV14-1*01_TRAJ26*01 pair (3.02%) had the highest percentage in the Arm group, but the TRAV13D-1*0_TRAJ32*01 pair (3.68%) had the highest percentage in the Cl13 group (**Supplementary Figure S5B**). Among the α-β chain VJ pairing profiles, the most common V(D)J pairing profile in expanded clones was TRAV6D-7*04_TRAJ23*01_TRBV15*01_TRBJ2-1*01 (2.89%), which accounted for a very high proportion of the clonotypes of Gzma-high effector-like CD8^+^ T cells in the Arm group, whereas TRAV4D-3*03_TRAJ43*01_TRBV13-2*01_TRBJ2-7*01 (3.51%) was the most common V(D)J pairing profile in the Cl13 group (**Figure 6H**).

Additionally, we evaluated the expression of TRBV and TRAV genes in Ifng-high effector-like CD8^+^ T cells from the Arm and Cl13 groups on 8 dpi. Our data showed high levels of TRBV20*01, TRBV13-1*02 and TRBV15*01 expression in Ifng-high effector-like CD8^+^ T cells from the Arm group but high levels of TRBV13-2*01 and TRBV5*01 expression in the Cl13 group (**Figure 6F**). Among the β chain VJ pairs, the TRBV20*01_TRBJ1-6*01 pair (8.33%) had the highest percentage in Ifng-high effector-like CD8^+^ T cells in the Arm group, but the TRBV5*01_TRBJ1-5*01 pair (11.89%) had the highest percentage in the Cl13 group (**Figure 6G**). Our data also showed high levels of TRAV12D-2*02 and TRAV6-5*04 expression in Ifng-high effector-like CD8^+^ T cells from the Arm group but high levels of TRAV4D-1*01, TRAV16D/DV11*03 and TRAV5-4*01 expression in the Cl13 group (**Supplementary Figure S5C**). Among the α chain VJ pairs, the TRAV12D-2*02_TRAJ22*01 pair (8.73%) had the highest percentage in Arm group, but the TRAV14D-1*01_TRAJ32*01 pair (10.96%) had the highest percentage in the Cl13 group (**Supplementary Figure S5D**). Among the α-β chain VJ pairing profiles, the most common V(D)J pairing profile in expanded clones was found to be TRAV12D-2*02_TRAJ22*01_TRBV20*01_TRBJ1-6*01 (8.8%), which accounted for a very high proportion of the clonotypes of Ifng-high effector-like CD8^+^ T cells in Arm group. However, it was found to be TRAV14D-1*01_TRAJ32*01_TRBV5*01_TRBJ1-5*01 (10.9%) in Cl13 group (**Figure 6I**).

### The clonal diversity of CD4^+^ T cells revealed by scTCR-seq during acute and chronic infections

**Figure 7:**
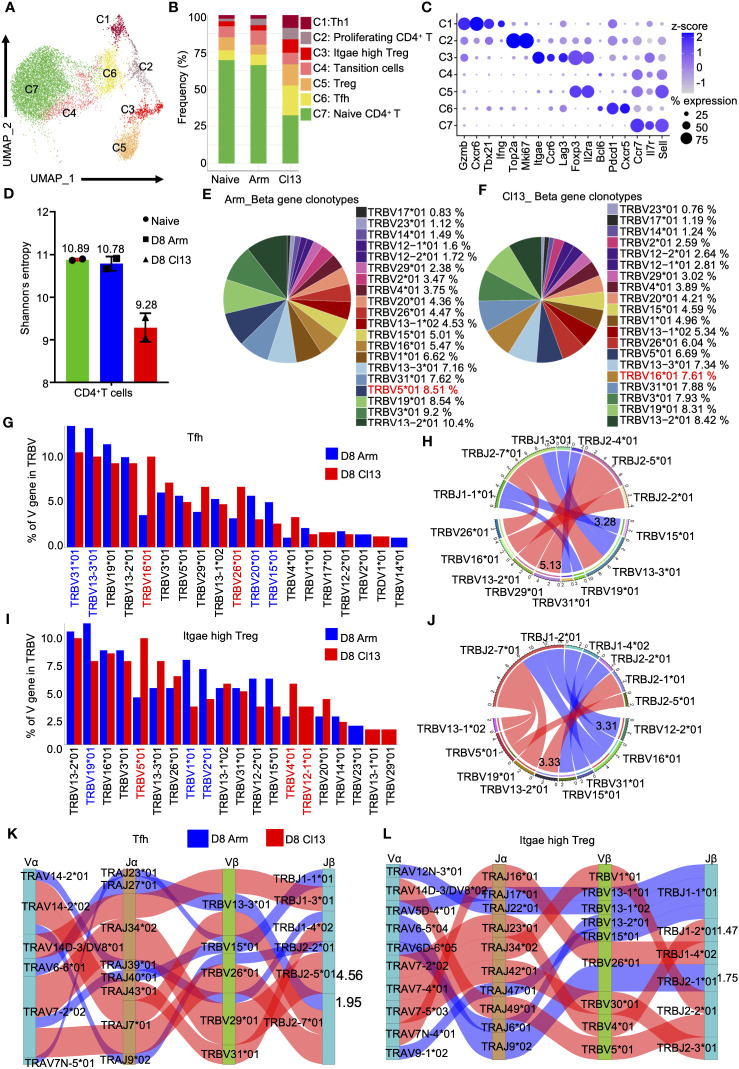
The clonal diversity of CD4^+^ T cell revealed by scTCR-seq during acute and chronic infection. **(A)** UMAP plot of CD4^+^ T cells from days 0 and 8 during Arm (LCMV-Armstrong) and Cl13 (LCMV-Cl13) infections. Cells are colored by T cells subtype as shown in legend on right. **(B)** Proportion of each defined cell type across groups. **(C)** Dot plot of marker genes for distinct cell types. Color scale indicates the mean of normalized expression of marker genes in each cell type, and dot size is proportional to the percentage of cells within each cell cluster expressing the marker genes. Cell cluster IDs on the left correspond to those in **(B)**. **(D)** Analysis of the clonal expended TCR β chain. Bar graphs showing the clonal diversity of CD4^+^ T cells from Arm and Cl13 (8 dpi), which was calculated using Shannon’s entropy. Pie chart showing the usage top 20 of TCR heavy chain V genes in CD4^+^ T cells from the Arm **(E)** and Cl13 **(F)** groups, respectively. **(G)** The bar plots showing usage of some V genes from Tfh cells TCR β chain of Arm group (blue)and Cl13 group (red) (8 dpi) (Top20). **(H)** Circos plots show the differential TCR β VJ pairs in Tfh cells from the Arm and Cl13 groups. Blue links represent acute groups specific VJ pairs, and red links represent chronic groups specific VJ pairs. **(I)** The bar plots showing usage of some V genes from Itgae high Treg cells TCR β chain of Arm group (blue)and Cl13 group (red) (8 dpi) (Top20). **(J)** Circos plots show the differential TCR β VJ pairs in Itgae high Treg cells from the Arm and Cl13 groups. Blue links represent acute groups specific VJ pairs, and red links represent chronic groups specific VJ pairs. **(H)** Sankey diagram shows significant different frequency of α-β VJ pairs in Tfh cells **(K)** and Itgae high Treg **(L)** from the Arm and Cl13 groups. Blue links represent Arm groups specific pairs, and red links represent Cl13 groups specific pairs.

Considering the failure to define well-recognized subpopulations of CD4^+^ T cells by scRNA-seq analysis (28), we simultaneously performed single-cell protein and RNA sequencing analyses, which integrated scRNA-seq analysis and application of AbSeq Antibody-Oligo Conjugates (see Methods). We identified 7 distinct subtypes (**Figures 7A, B**) based on their respective gene expression profiles. Clusters corresponding to naive CD4^+^ T, Tfh, Treg, transition, *Itgae*-high Treg, proliferating CD4^+^ T, Tfh, and Th1 cells were identified based on known markers (**Figures 7A, C**). These markers mainly included *Sell*, *Il7r*, and *Ccr7* for naive CD4^+^ T cells; *Cxcr5*, *Pdcd1*, and *Bcl6* for Tfh cells; *Il2ra*, and *Foxp3* for Treg cells; *Il2ra*, *Foxp3*, *Lag3*, *Ccr6*, and *Itgae* for *Itgae*-high Treg cells; proliferating CD4^+^ T cells; and *Ifng*, *Tbx21*, *Cxcr6*, and *Gzmb* for Th1 cells (**Figure 7C**).Indeed, we found more conventional Treg cells, *Itgae*-high Treg cells and Tfh cells during chronic infection(**Figure 7B**.).

Then, we quantified the diversity of CD4^+^ T-cell TCR repertoire using the Shannon entropy index. The higher the Shannon entropy index is, the more diverse the distribution of CDR3 clones. For CD4^+^ T cells, entropy index of the uninfected sample was 10.89, whereas index for Arm group was 10.78 and that for the Cl13 group was 9.28 (**Figure 7D**). These results showed the reduced diversity of CD4^+^ T cells during chronic infection. In addition, we analyzed the usage of top 5 TCR heavy chain V genes in CD4^+^ T cells from the acute and chronic infection groups and found that each group had one V gene that was different from the other group: TRBV5 * 01 (8.51%) in the acute infection group (**Figure 7E**) and TRBV16*01 (7.61%) in the chronic infection group (**Figure 7F**).

Tfh cells help host humoral immunity to control chronic viral infection by promoting the production of antibodies (41). Our data showed that the percentage of Tfh cells during chronic viral infection was significantly higher than that during acute viral infection (**Figure 7B**). Moreover, we evaluated the expression of TRBV and TRAV genes in Tfh cells from the Arm and Cl13 groups on day 8. Our data showed high levels of TRBV16*01, TRBV29*01 and TRBV26*01 expression in Cl13 compared with Arm group (**Figure 7G**). Our data also showed high levels of TRAV6−6*01 and TRAV14−2*01 expression in Tfh cells from the Arm group but high levels of TRAV14D−3/DV8*01, TRAV7−2*02 and TRAV14−2*02 expression in the Cl13 group (**Supplementary Figure S5E**). Among the β chain VJ pairs, the TRBV15*01_TRBJ1-1*01 pair (3.28%) had the highest percentage in the Arm group, but the TRBV29*01_TRBJ2-5*01 pair (5.13%) had the highest percentage in the Cl13 group (**Figure 7H**). Among the α chain VJ pairs, the TRAV6-6*01_TRAJ27*01 pair (2.19%) had the highest percentage in the Arm group, but the TRAV7-2*02_TRAJ34*02 pair (4.42%) had the highest percentage in the Cl13 group (**Supplementary Figure S5F**). Among the α-β chain VJ pairing profiles, the most common V(D)J pairing profile in expanded clones was found to be TRAV6-6*01_TRAJ27*01_TRBV13-3*01_TRBJ2-7*01 (1.95%), which accounted for a very high proportion of the clonotypes of Tfh cells in Arm, whereas in Cl13 group, it was found to be TRAV7-2*02_TRAJ34*02_TRBV29*01_TRBJ2-5*01 (4.56%) (**Figure 7K**).

CD103^+^ (encoded by *Itgae*) Treg cells are more potent inhibitors of T-cell proliferation than conventional Treg cells (42, 43). Moreover, our data showed that the percentage of Itgae-high Treg cells during chronic viral infection was significantly higher than that during acute viral infection (**Figure 7B**). Thus, we evaluated the expression of TRBV and TRAV genes in Itgae-high Treg cells from the Arm and Cl13 groups on Day 8. Our data showed that there were high levels of TRBV19*01, TRBV1*01 and TRBV2*01 expression in Arm compared to Cl13 group but high levels of TRBV5*01, TRBV13-3*01 and TRBV4*01 expression in Cl13 group (**Figure 7I**).

Our data also showed high levels of TRAV6−5*04, TRAV4D−4*<02no></no> and TRAV5D−4*01 expression in Itgae-high Treg cells from the Arm group but high levels of TRAV14−3*01, TRAV7−2*02, TRAV14−2*02 and TRAV6−3*01 expression in the Cl13 group (**Supplementary Figure S5G**). Among the β chain VJ pairs, TRBV12-2*01_TRBJ2-7*01 pair (3.31%) exhibited increased usage in Arm group compared with Cl13 group, but TRBV13-2*01_TRBJ2-1*01 pair (3.33%) had the highest percentage in the Cl13 group (**Figure 7J**). Among the α chain VJ pairs, the TRAV4D-4*03_TRAJ12*01 pair (2.27%) had the highest percentage in the Arm group, but the TRAV7-2*02_TRAJ23*01 pair (1.82%) had the highest percentage in the Cl13 group (**Supplementary Figure S5H**). Among the α-β chain VJ pairing profiles, the V(D)J pairing profile with the highest frequency in expanded clones was found to be TRAV6-5*04_TRAJ9*02_TRBV26*01_TRBJ2-1*01 (1.75%), which accounted for a very high proportion of the clonotypes of Itgae-high Treg cells in Arm group. However, it was found to be TRAV7-2*02_TRAJ23*01_TRBV4*01_TRBJ1-2*01 (1.47%) in the Cl13 group (**Figure 7L**).

## Discussion

The comparative landscape of immune cells responding to acute and chronic viral infections remains largely unclear. In this study, to explore the dynamics and heterogeneity of immune cells in acute and chronic viral infections at different times, we analyzed CD45^+^ immune cells from mouse lymph nodes using high-throughput scRNA-seq, single-cell B-cell receptor sequencing (scBCR-seq) and single-cell T-cell receptor sequencing (scTCR-seq).

The host control of acute and chronic viral infections requires the activation of innate cells to initiate and maintain adaptive immune responses (44–46). In this study, NK cells exhibited a higher frequency during chronic infection than acute infection on 4 dpi. Afterward, the proportion of NK cells decreased comparably in the two groups. Furthermore, GO enrichment analysis and GSEA showed that NK cells were functionally impaired in response to type I interferon or chronic viral infection. Unlike NK cells, innate immune myeloid cells were subsequently expanded at the acute effector stage (8 dpi), and their proportion was lower during chronic infection.

Dendritic cells, including conventional DCs (cDCs) that act as antigen-presenting cells and plasmacytoid DCs (pDCs) that produce type I interferons, mediated innate and adaptive antiviral responses (47, 48). The dynamics of dendritic cells in acute and chronic viral infections remain unclear. In this study, compared with that in acute infection, pDCs significantly decreased at the acute effector stage (8 dpi) and exhibited significantly higher enrichment of cytokine activity in chronic infection. Toll-like receptor 7(TLR7) is an innate signaling receptor that is primarily expressed by pDCs (49). Previous research has shown that TLR7 signaling dictates the establishment of chronic LCMV Cl13 but does not affect the clearance of the acute LCMV Arm strain (47). Our results showed that pDCs in chronic infection exhibited significantly higher enrichment of Toll-like receptor signaling pathways. However, the functional change of pDCs in chronic viral infection remains to be investigated considering the unknown functional roles of DC in viral infection (50, 51).

The humoral immune response mediated by B cells plays a critical role in host defense against a variety of pathogens through secreting antibodies by plasma cells (52, 53). In this study, chronic infection elicited more robust germinal center B-cell responses and antibody production than acute infection, consistent with previous studies (11). Surprisingly, the proportion of MALT B cells in chronic infection was significantly higher than that in acute infection on 8 dpi.

The B cells undergo rapid clonal expansion and somatic hypermutation in germinal centers (GCs) (54). With the help of Tfh cells in the light zone of GCs, B cells differentiate into high-affinity antibody-secreting plasma cells and resultant memory B cells (55, 56). Consistent with previous studies (11), our study showed that chronic infection induces a more robust germinal center B-cell response and higher production of antibody-producing plasma cells than acute infection. Overall, the B-cell immune response was more intense in chronic infection; however, it failed to clear the viruses once persistent infection was established, which deserves further exploration. Of note, we also found plasma cells expressed different dominant BCRs in acute and chronic viral infections.

CD8^+^ T cells play essential roles in specific defense against viral infections (12, 57). The dynamics of CD8^+^ T cells in lymphoid tissues during acute and chronic LCMV infections remain unclear. In this study, we identified two CD8^+^ T cell subsets that remarkedly changed throughout LCMV infection: Gzma-high effector-like CD8^+^ T cells and Ifng-high effector-like CD8^+^ T cells. Unlike acute infection, Gzma-high effector-like CD8^+^ T cells were more highly induced in the early stage of chronic infection, indicating that they may be involved in the establishment of persistent infection. However, proportion of *Ifng*-high effector-like CD8^+^ T cells also increased with the time since infection, peaking on 8 dpi. Then, on 30 dpi, the proportions approached the baseline levels.

Unlike acute infection or vaccination, where antigen is cleared soon and naive CD8^+^ T cells differentiate into functional effector cells and subsequent memory cells, persistent antigen exposure leads to CD8^+^ T cell exhaustion (7, 58). In contrast to classical memory CD8^+^ T cells, exhausted-like CD8^+^ T cells are characterized by sustained expression of inhibitory receptors, such as programmed cell death protein 1 (PD-1), LAG-3, 2B4, CD160, and *Havcr2* (TIM-3), and impaired effector functions (12) (59–61). In our study, *Ifng*-high effector-like CD8^+^ T cells also transcriptionally expressed *Pdcd1*, *Havcr2* and *Lag3*. However, exhausted CD8^+^ T cells also transcriptionally expressed a high level of *Ifng*, where the implication needs to be further revealed. Of note, the dominant clonotypes of TCRs in both Gzma-high effector-like and Ifng-high effector-like CD8^+^ T cells were differentially induced in acute and chronic infections. In addition, consistent with the conclusion that TCR repertoire contracts over time in chronic infection (62), our data also showed narrower TCR diversity in chronic LCMV infection.

CD4^+^ T cells during chronic infection are heterogeneous and display developmental plasticity (18). Considering the failure to clearly define CD4^+^ T cell subpopulations by scRNA-seq analysis (28), we resequenced and analyzed CD4^+^ cells by integrating single-cell RNA-seq and single-cell antibody sequencing. Herein, we identified 7 distinct CD4^+^ T subtypes, including naive CD4^+^ T, Tfh, Treg, transition, Itgae-high Treg, proliferating CD4^+^ T, and Th1 cells. Multiple CD4^+^ T subtypes were induced higher in chronic viral infection, indicating overall activation of CD4^+^ T cells in chronic viral infection. Furthermore, the diversity of TCR usage was weakened in chronic viral infection from virus-specific CD4^+^ T cells (18).

Tfh cells provide essential support for humoral immunity by promoting the production of antibodies during viral infection (41). Chronic viral infection is associated with Th1 dysfunction and a skewed shift to Tfh differentiation (63). Consistently, the percentage of Tfh cells during chronic viral infection was significantly higher than that during acute viral infection, which was associated with robust B cell response. However, the protective roles of Tfh and B cells in chronic viral infection need further investigation. Intriguingly, the TCRs in Tfh cells were also differentially used between acute and chronic infection.

CD103 (*Itgae*)^+^ Treg cells exert more potent inhibitory functions than conventional Treg cells (42, 43). Moreover, our data showed that the percentage of Itgae-high Treg cells during chronic viral infection was significantly higher than that during acute viral infection. Again, acute and chronic LCMV infection induced different dominant TCRs in this population.

Although the comprehensive comparison of immune cells between acute and chronic viral infections was described, there still are some limitations in this study. Although the GO and GSEA data is partially supported by other methodologies, the validation of enrichment of specific genes of some immune subsets (such as Ifng high effector-like CD8^+^ T cells) is still needed. Regarding the immune responses to viral infection in humans, conclusions in this study based on animal models could only be referred to and need further validation in clinical practices. In addition, this study could not provide measures for clinically treating the acute viral infection or preventing the persistence of viruses, considering the complicated immune response to viral infection in humans and the strict requirements for clinical usage.

In conclusion, the longitudinal dynamics and heterogeneity of lymph node immune cells during acute and chronic viral infections were revealed by utilizing scRNA-seq, scTCR-seq, and scBCR-seq analysis. In brief, chronic viral infection induced faster and more robust NK cells, and exerted decreased but aberrantly activated pDCs at the acute phase. Simultaneously, there were significantly increased IgA^+^ plasma cells (MALT B cells) but differential usage of B-cell receptors in chronic infection. Regarding T-cell responses, Gzma-high effector-like CD8^+^ T cells were aberrantly activated and accompanied by temporally reversed gene expression profiles throughout viral infection. Chronic infection also induced more robust CD4^+^ T cell responses. In addition, chronic infection compromised TCR diversity in both CD8^+^ and CD4^+^ T cells but showed differential usage of most dominant TCR clonotype. Thus, this study provides new insights into longitudinal maps of immune cells during acute and chronic viral infections and clarifies transcriptional profiles and TCR/BCR repertoires of these cells after viral infection.

## Data availability statement

The datasets presented in this study can be found in online repositories. The names of the repository/repositories and accession number(s) can be found below: PRJNA928568 and PRJNA922879 (SRA).

## Ethics statement

The animal study was approved by The Animal Ethical and Welfare Committee of Shenzhen University (IACUC-202300026). The study was conducted in accordance with the local legislation and institutional requirements.

## Author contributions

YJ: Investigation, Methodology, Writing – original draft, Formal Analysis, Visualization. YH: Methodology, Writing – review & editing, Formal Analysis, Visualization. BL: Methodology, Writing – review & editing. XZ: Methodology, Writing – review & editing. CS: Methodology, Writing – review & editing. YW: Methodology, Writing – review & editing. WH: Methodology, Writing – review & editing. YY: Methodology, Writing – review & editing. NC: Methodology, Writing – review & editing. YD: Methodology, Writing – review & editing. YO: Methodology, Writing – review & editing. YW: Methodology, Writing – review & editing. MZ: Methodology, Writing – review & editing. SX: Conceptualization, Supervision, Writing – review & editing, Funding acquisition.
