# Supplementary material for: Single-cell RNA sequencing reveals the dynamics and heterogeneity of lymph node immune cells during acute and chronic viral infections
Source: Front Immunol. 2024 Jan 29;15:1341985. doi: 10.3389/fimmu.2024.1341985 (PMC10863051; doi:10.3389/fimmu.2024.1341985)
Supplement: Supplementary file 1 [file DataSheet_1.pdf]

Figure. S1

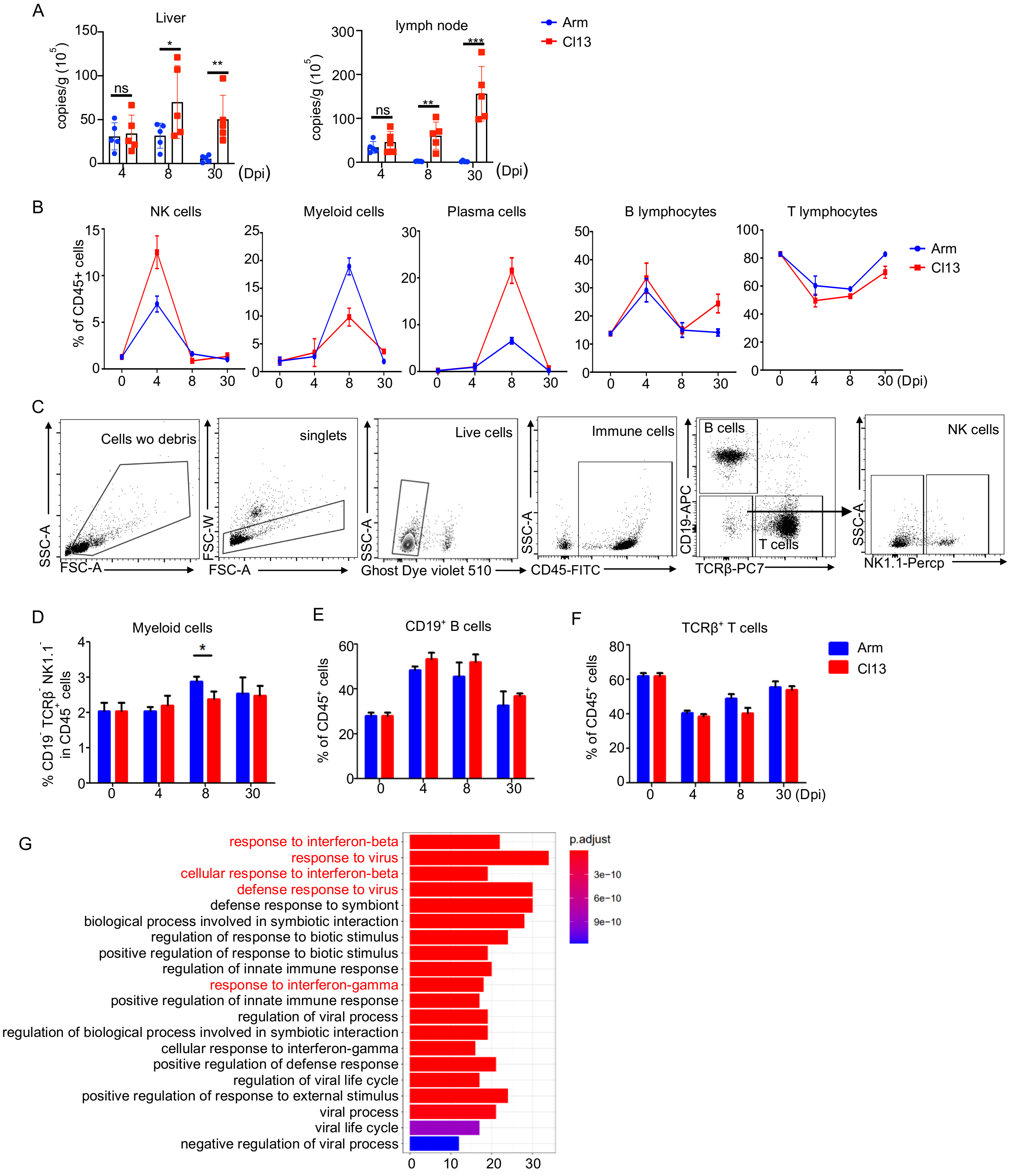
A
Liver
copies/g (10^5)
150
100
50
0
ns
*
**
4
8
30
(Dpi)
lymph node
300
200
100
0
ns
**
***
Arm
Cl13
B
NK cells
Myeloid cells
Plasma cells
B lymphocytes
T lymphocytes
% of CD45+ cells
30 (Dpi)
C
Cells wo debris
singlets
Live cells
Immune cells
B cells
T cells
NK cells
SSC-A
FSC-A
FSC-W
Ghost Dye violet 510
CD45-FITC
CD19-APC
TCRβ-PC7
NK1.1-Percp
D
Myeloid cells
% CD19- TCRβ- NK1.1- in CD45+ cells
*
E
CD19+ B cells
% of CD45+ cells
F
TCRβ+ T cells
% of CD45+ cells
30 (Dpi)
G
response to interferon-beta
response to virus
cellular response to interferon-beta
defense response to virus
defense response to symbiont
biological process involved in symbiotic interaction
regulation of response to biotic stimulus
positive regulation of response to biotic stimulus
regulation of innate immune response
response to interferon-gamma
positive regulation of innate immune response
regulation of viral process
regulation of biological process involved in symbiotic interaction
cellular response to interferon-gamma
positive regulation of defense response
regulation of viral life cycle
positive regulation of response to external stimulus
viral process
viral life cycle
negative regulation of viral process
0
10
20
30
p.adjust
3e−10
6e−10
9e−10

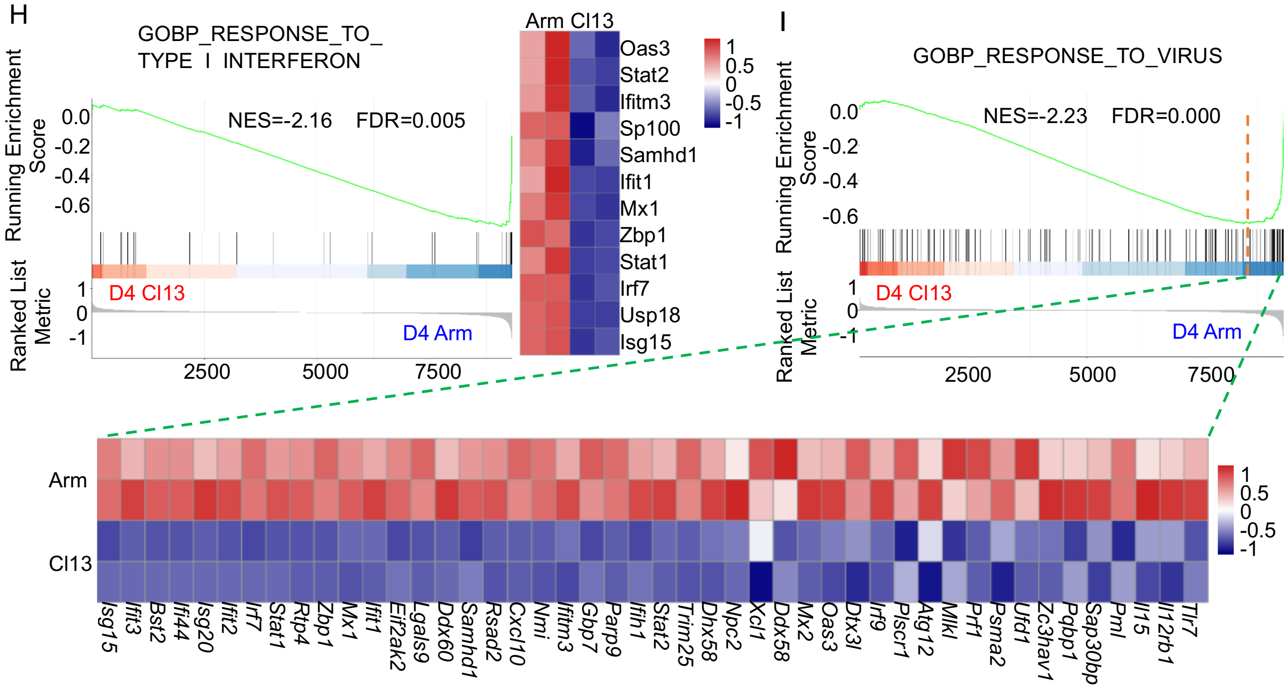
H
GOBP_RESPONSE_TO_
TYPE I INTERFERON
NES=-2.16 FDR=0.005
Running Enrichment Score
0.0
-0.2
-0.4
-0.6
Ranked List Metric
1
0
-1
D4 Cl13
D4 Arm
2500
5000
7500
Arm Cl13
Oas3
Stat2
Ifitm3
Sp100
Samhd1
Ifit1
Mx1
Zbp1
Stat1
Irf7
Usp18
Isg15
1
0.5
0
-0.5
-1
I
GOBP_RESPONSE_TO_VIRUS
NES=-2.23 FDR=0.000
Running Enrichment Score
0.0
-0.2
-0.4
-0.6
Ranked List Metric
1
0
-1
D4 Cl13
D4 Arm
2500
5000
7500
Arm
Cl13
Isg15
Ifit3
Bst2
Ifi44
Isg20
Ifit2
Irf7
Stat1
Rtp4
Zbp1
Mx1
Ifit1
Eif2ak2
Lgals9
Ddx60
Samhd1
Rsad2
Cxcl10
Nmi
Ifitm3
Gbp7
Parp9
Ifih1
Stat2
Trim25
Dhx58
Npc2
Xcl1
Ddx58
Mx2
Oas3
Dtx3l
Irf9
Plscr1
Atg12
Mlkl
Prf1
Psma2
Ufd1
Zc3hav1
Pqbp1
Sap30bp
Pml
Il15
Il12rb1
Tlr7
1
0.5
0
-0.5
-1

Figure.S1. (A) The viral load of liver and lymph nodes of mice infected with virus for days 4, 8 and 30 was detected by quantitative RT-PCR analysis for the LCMV-GP RNA. (n = 5 for each group). (B) Percentage of each identified cell subtype NK cells, Myeloid cells, B lymphocytes and T lymphocytes) across groups during Arm (LCMV-Armstrong) and Cl13 (LCMV-Cl13) infections from Fig. 1 D. (C) Gating strategy for the analysis of B cells, T cells, NK cells and myeloid cells by flow cytometry. Flow cytometry of myeloid cells, $CD19^{+}$ B cells and $TCR\beta^{+}$ T cells in lymph nodes of mice infected with the virus for days 0, 4, 8 and 30. Plots are gated on $CD45^{+}$ cells. The percentages of the myeloid cells, $CD19^{+}$ B cells and $TCR\beta^{+}$ T cells were shown in representative Dot Plot and (D, E and F) cumulative data on the frequency were summarized in bar graphs (n = 6 from 2 independent experiments). (G) GO BP enrichment analysis of differentially expressed genes downregulated about D8 Cl13 vs. Arm in NK cells. The top 20 enriched GO terms are shown. GO rich column-shaped diagram. The vertical coordinates were rich GO terms, and the horizontal coordinates were the number of different genes in the term. (H and I) GSEA showed enriched expression of genes in the NK cells derived from D8 Cl13 infection and Arm infection group, with the enriched genes displayed in a heatmap. NES, normalized ES; FDR, false discovery rate. ***,P<0.001; **,P<0.01; *,P<0.05; ns, P > 0.05. Statistical significance in A and D was determined Student's t test for indicated pairwise comparisons.

Figure.S2

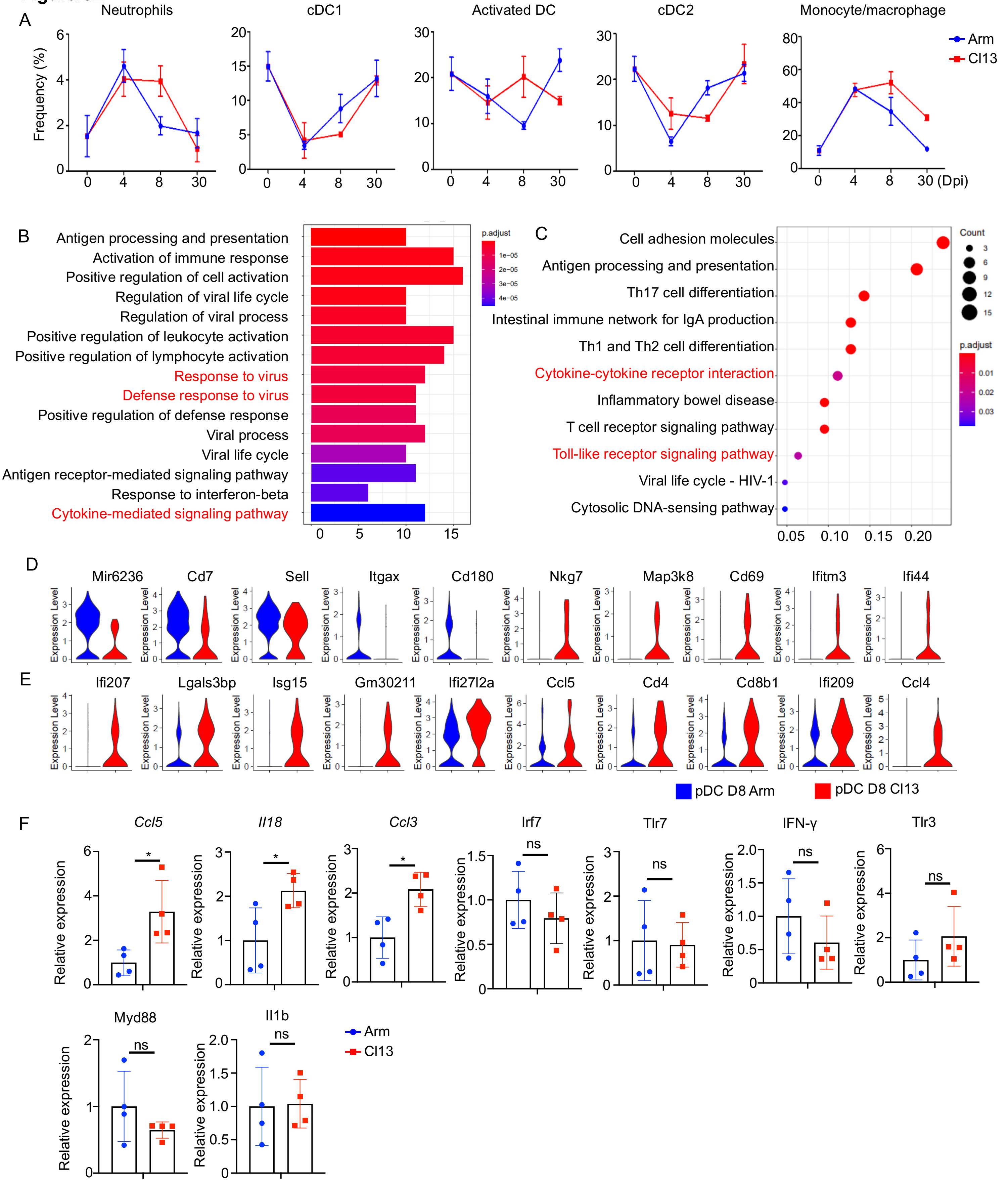
A
Neutrophils
cDC1
Activated DC
cDC2
Monocyte/macrophage
Frequency (%)
0
4
8
30
30 (Dpi)
Arm
Cl13
B
Antigen processing and presentation
Activation of immune response
Positive regulation of cell activation
Regulation of viral life cycle
Regulation of viral process
Positive regulation of leukocyte activation
Positive regulation of lymphocyte activation
Response to virus
Defense response to virus
Positive regulation of defense response
Viral process
Viral life cycle
Antigen receptor-mediated signaling pathway
Response to interferon-beta
Cytokine-mediated signaling pathway
p.adjust
1e-05
2e-05
3e-05
4e-05
C
Cell adhesion molecules
Antigen processing and presentation
Th17 cell differentiation
Intestinal immune network for IgA production
Th1 and Th2 cell differentiation
Cytokine-cytokine receptor interaction
Inflammatory bowel disease
T cell receptor signaling pathway
Toll-like receptor signaling pathway
Viral life cycle - HIV-1
Cytosolic DNA-sensing pathway
Count
p.adjust
0.01
0.02
0.03
0.05
0.10
0.15
0.20
D
Mir6236
Cd7
Sell
Itgax
Cd180
Nkg7
Map3k8
Cd69
Ifitm3
Ifi44
Expression Level
E
Ifi207
Lgals3bp
Isg15
Gm30211
Ifi27l2a
Ccl5
Cd4
Cd8b1
Ifi209
Ccl4
pDC D8 Arm
pDC D8 Cl13
F
Ccl5
Il18
Ccl3
Irf7
Tlr7
IFN-γ
Tlr3
Myd88
Il1b
Relative expression
*
ns
Arm
Cl13

Figure.S2. (A)Percentage of each identified cell subtype (Neutrophils, monocytes/macrophages, cDC1, cDC2 and activated DC) within myeloid cells across groups during Arm (LCMV-Armstrong) and Cl13 (LCMV-Cl13) infections from Fig. 2 B. Gene Ontology (GO) (B) and (C) Kyoto Encyclopedia of Genes and Genomes (KEGG) enrichment analysis was performed using differentially expressed genes (DEGs) in pDCs from Cl13 infection group vs. Arm infection group on day 8. The top 20 enriched GO terms are shown. (B) GO rich column-shaped diagram. The vertical coordinates were rich GO terms, and the horizontal coordinates were the number of different genes in the term. (C) KEGG enrichment analysis was shown in scattered dots. The vertical axis indicates the pathway name, the horizontal axis represents Rich Factor, and the size of the point indicates the number of genes in this pathway, and the color of the point corresponds to different p.adjust ranges. (D and E) Violin plots showing the expressing profile of differential genes in pDCs from Cl13 infection group and Arm infection group on day 8. (F) The different genes in pDCs from Cl13 infection group and Arm infection group on day 8 were analyzed by qPCR. The expression of each gene in pDCs from Arm infection group mice was set at 1, and that in pDCs from Cl13 infection group was normalized accordingly. (n = 4 for each group).*, $P<0.05$; ns,$P > 0.05$. Statistical significance in F was determined Student's t test for indicated pairwise comparisons.

Figure.S3

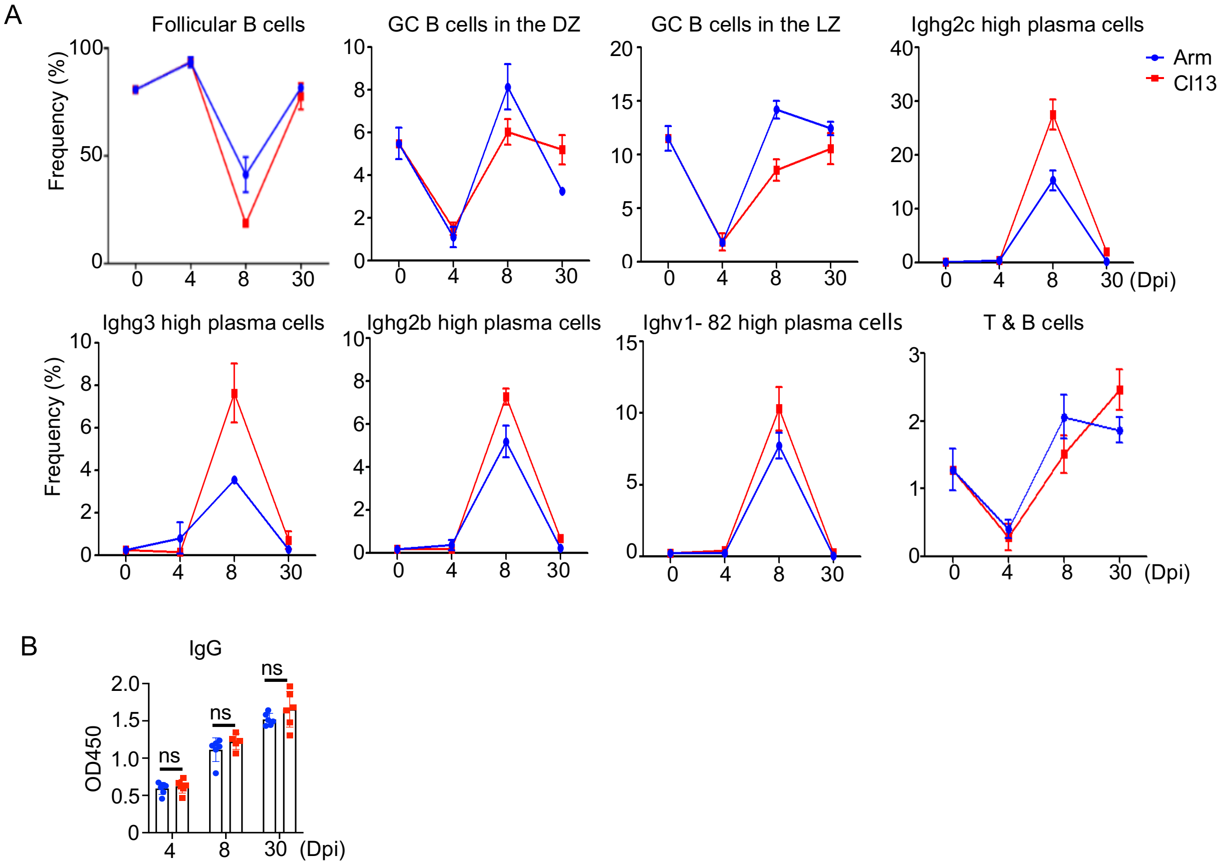

Figure.S3. (A) Percentage of each identified cell subtype (Follicular B cells, GC B cell in DZ, GC B cell in LZ, Ighg2c high plasma cells, MALT B cells, Ighg2b high plasma cells, Ighg3 high plasma cells and Ighv1−82 high plasma cells) across groups during Arm (LCMV-Armstrong) and Cl13 (LCMV-Cl13) infections from Fig. 3 B. (B) The secretion of IgG was analyzed by ELISA. n = 6 for each group. ns, $P > 0.05$. Statistical significance in B was determined by by Student's t test for indicated pairwise comparisons.

Figure.S4

A

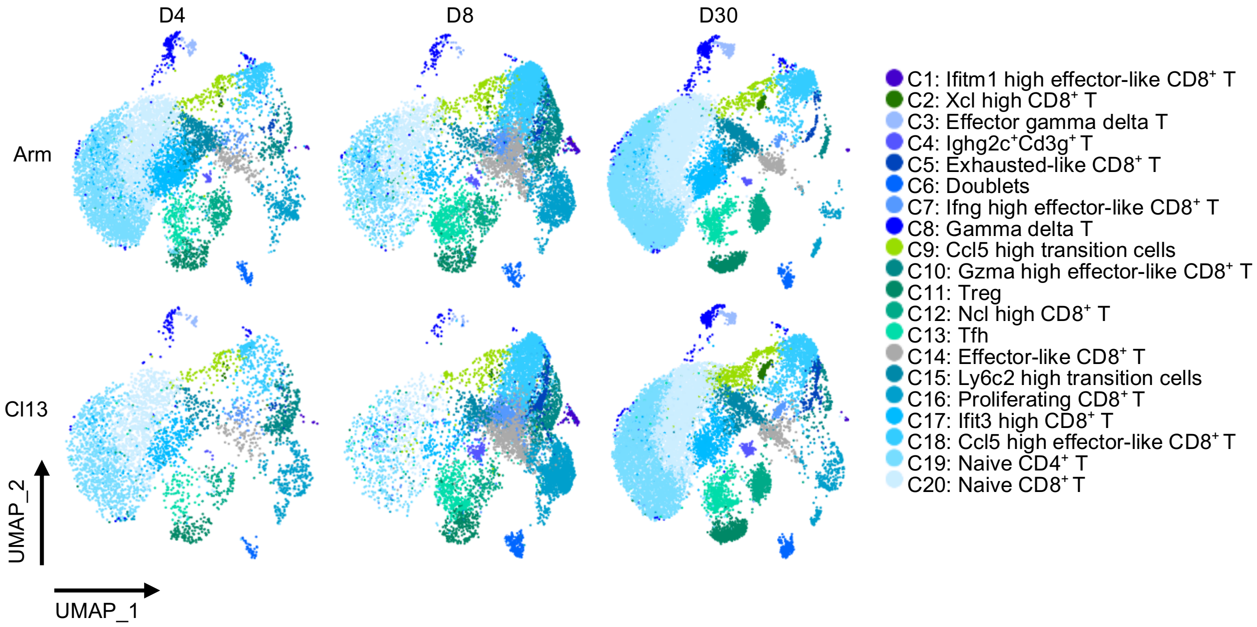
D4
D8
D30
Arm
Cl13
UMAP_2
UMAP_1
C1: Ifitm1 high effector-like CD8⁺ T
C2: Xcl high CD8⁺ T
C3: Effector gamma delta T
C4: Ighg2c⁺Cd3g⁺ T
C5: Exhausted-like CD8⁺ T
C6: Doublets
C7: Ifng high effector-like CD8⁺ T
C8: Gamma delta T
C9: Ccl5 high transition cells
C10: Gzma high effector-like CD8⁺ T
C11: Treg
C12: Ncl high CD8⁺ T
C13: Tfh
C14: Effector-like CD8⁺ T
C15: Ly6c2 high transition cells
C16: Proliferating CD8⁺ T
C17: Ifit3 high CD8⁺ T
C18: Ccl5 high effector-like CD8⁺ T
C19: Naive CD4⁺ T
C20: Naive CD8⁺ T

B

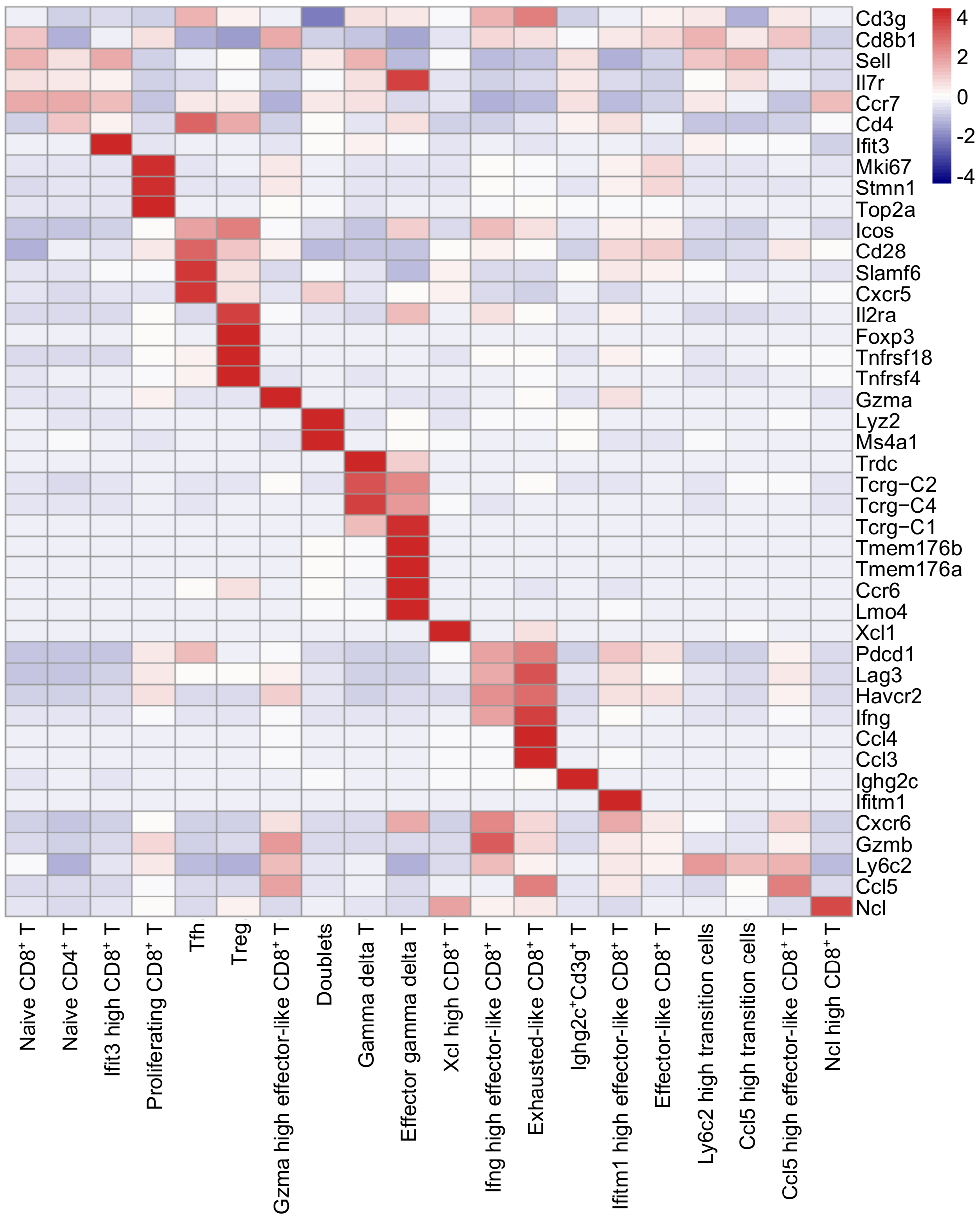
Cd3g
Cd8b1
Sell
Il7r
Ccr7
Cd4
Ifit3
Mki67
Stmn1
Top2a
Icos
Cd28
Slamf6
Cxcr5
Il2ra
Foxp3
Tnfrsf18
Tnfrsf4
Gzma
Lyz2
Ms4a1
Trdc
Tcrg−C2
Tcrg−C4
Tcrg−C1
Tmem176b
Tmem176a
Ccr6
Lmo4
Xcl1
Pdcd1
Lag3
Havcr2
Ifng
Ccl4
Ccl3
Ighg2c
Ifitm1
Cxcr6
Gzmb
Ly6c2
Ccl5
Ncl
4
2
0
-2
-4
Naive CD8⁺ T
Naive CD4⁺ T
Ifit3 high CD8⁺ T
Proliferating CD8⁺ T
Tfh
Treg
Gzma high effector-like CD8⁺ T
Doublets
Gamma delta T
Effector gamma delta T
Xcl high CD8⁺ T
Ifng high effector-like CD8⁺ T
Exhausted-like CD8⁺ T
Ighg2c⁺Cd3g⁺ T
Ifitm1 high effector-like CD8⁺ T
Effector-like CD8⁺ T
Ly6c2 high transition cells
Ccl5 high transition cells
Ccl5 high effector-like CD8⁺ T
Ncl high CD8⁺ T

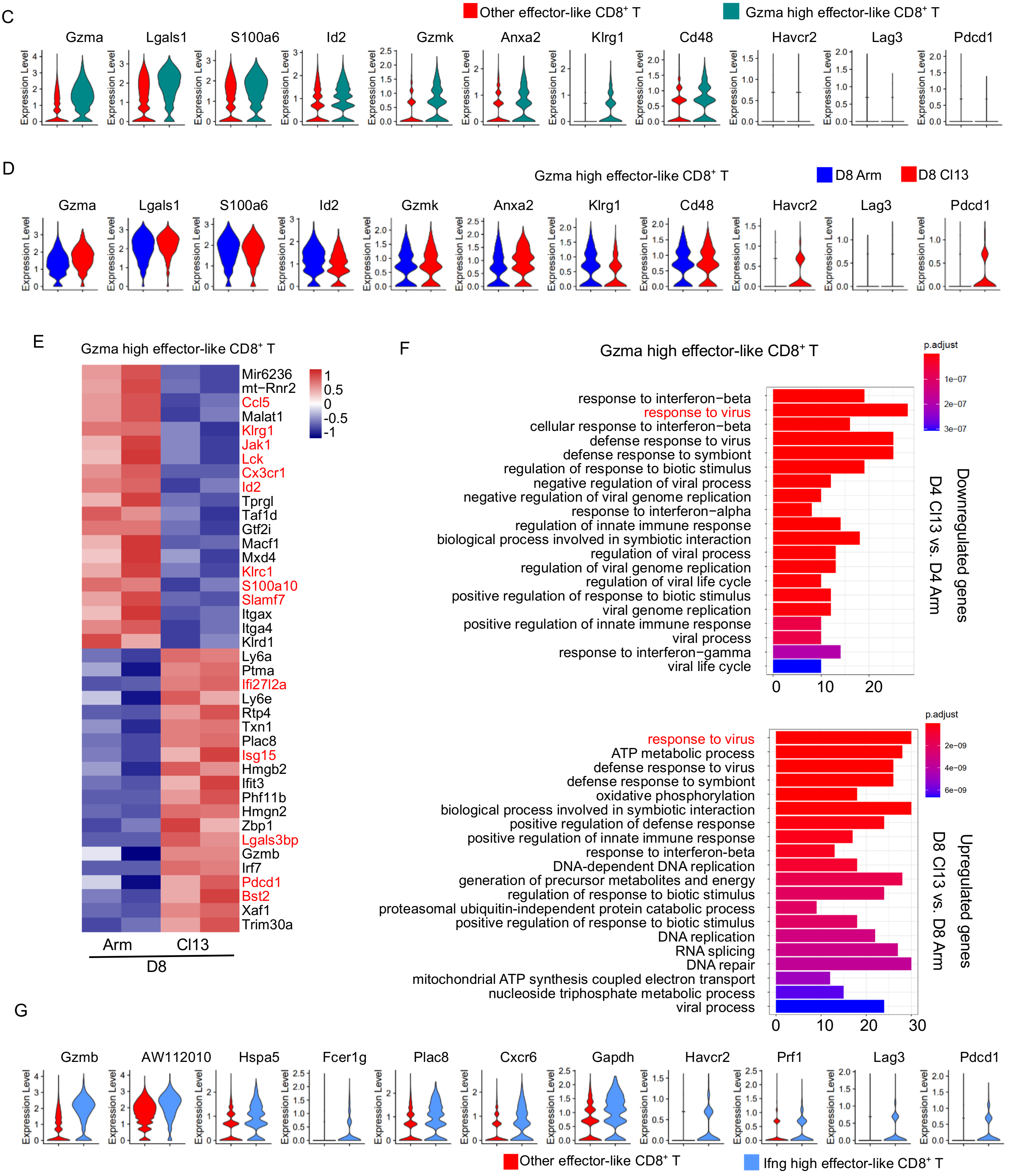

C
Other effector-like CD8+ T
Gzma high effector-like CD8+ T
Gzma Lgals1 S100a6 Id2 Gzmk Anxa2 Klrg1 Cd48 Havcr2 Lag3 Pdcd1
Expression Level
D
Gzma high effector-like CD8+ T
D8 Arm
D8 Cl13
Gzma Lgals1 S100a6 Id2 Gzmk Anxa2 Klrg1 Cd48 Havcr2 Lag3 Pdcd1
Expression Level
E
Gzma high effector-like CD8+ T
Mir6236
mt−Rnr2
Ccl5
Malat1
Klrg1
Jak1
Lck
Cx3cr1
Id2
Tprgl
Taf1d
Gtf2i
Macf1
Mxd4
Klrc1
S100a10
Slamf7
Itgax
Itga4
Klrd1
Ly6a
Ptma
Ifi27l2a
Ly6e
Rtp4
Txn1
Plac8
Isg15
Hmgb2
Ifit3
Phf11b
Hmgn2
Zbp1
Lgals3bp
Gzmb
Irf7
Pdcd1
Bst2
Xaf1
Trim30a
1
0.5
0
-0.5
-1
Arm
Cl13
D8
F
Gzma high effector-like CD8+ T
p.adjust
1e-07
2e-07
3e-07
response to interferon−beta
response to virus
cellular response to interferon−beta
defense response to virus
defense response to symbiont
regulation of response to biotic stimulus
negative regulation of viral process
negative regulation of viral genome replication
response to interferon−alpha
regulation of innate immune response
biological process involved in symbiotic interaction
regulation of viral process
regulation of viral genome replication
regulation of viral life cycle
positive regulation of response to biotic stimulus
viral genome replication
positive regulation of innate immune response
viral process
response to interferon−gamma
viral life cycle
0 10 20
Downregulated genes
D4 Cl13 vs. D4 Arm
p.adjust
2e-09
4e-09
6e-09
response to virus
ATP metabolic process
defense response to virus
defense response to symbiont
oxidative phosphorylation
biological process involved in symbiotic interaction
positive regulation of defense response
positive regulation of innate immune response
response to interferon-beta
DNA-dependent DNA replication
generation of precursor metabolites and energy
regulation of response to biotic stimulus
proteasomal ubiquitin-independent protein catabolic process
positive regulation of response to biotic stimulus
DNA replication
RNA splicing
DNA repair
mitochondrial ATP synthesis coupled electron transport
nucleoside triphosphate metabolic process
viral process
0 10 20 30
Upregulated genes
D8 Cl13 vs. D8 Arm
G
Gzmb AW112010 Hspa5 Fcer1g Plac8 Cxcr6 Gapdh Havcr2 Prf1 Lag3 Pdcd1
Expression Level
Other effector-like CD8+ T
Ifng high effector-like CD8+ T

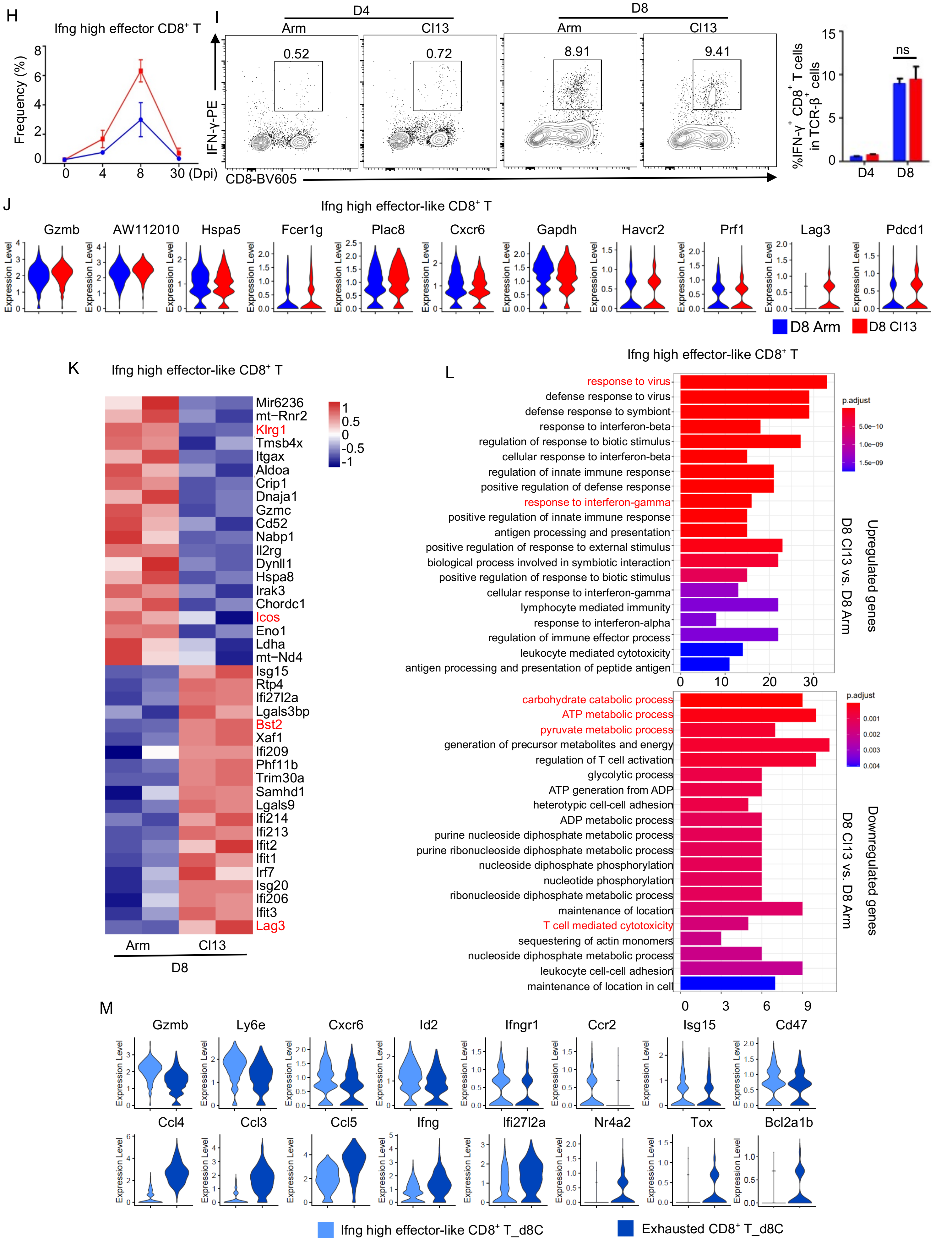
H
Ifng high effector CD8+ T
Frequency (%)
0 4 8 30 (Dpi)
I
D4
D8
Arm
Cl13
0.52
0.72
8.91
9.41
IFN-γ-PE
CD8-BV605
%IFN-γ+ CD8+ T cells in TCR-β+ cells
ns
D4
D8
J
Ifng high effector-like CD8+ T
Gzmb
AW112010
Hspa5
Fcer1g
Plac8
Cxcr6
Gapdh
Havcr2
Prf1
Lag3
Pdcd1
Expression Level
D8 Arm
D8 Cl13
K
Ifng high effector-like CD8+ T
Mir6236
mt−Rnr2
Klrg1
Tmsb4x
Itgax
Aldoa
Crip1
Dnaja1
Gzmc
Cd52
Nabp1
Il2rg
Dynll1
Hspa8
Irak3
Chordc1
Icos
Eno1
Ldha
mt−Nd4
Isg15
Rtp4
Ifi27l2a
Lgals3bp
Bst2
Xaf1
Ifi209
Phf11b
Trim30a
Samhd1
Lgals9
Ifi214
Ifi213
Ifit2
Ifit1
Irf7
Isg20
Ifi206
Ifit3
Lag3
Arm
Cl13
D8
L
Ifng high effector-like CD8+ T
response to virus
defense response to virus
defense response to symbiont
response to interferon-beta
regulation of response to biotic stimulus
cellular response to interferon-beta
regulation of innate immune response
positive regulation of defense response
response to interferon-gamma
positive regulation of innate immune response
antigen processing and presentation
positive regulation of response to external stimulus
biological process involved in symbiotic interaction
positive regulation of response to biotic stimulus
cellular response to interferon-gamma
lymphocyte mediated immunity
response to interferon-alpha
regulation of immune effector process
leukocyte mediated cytotoxicity
antigen processing and presentation of peptide antigen
p.adjust
D8 Cl13 vs. D8 Arm
Upregulated genes
carbohydrate catabolic process
ATP metabolic process
pyruvate metabolic process
generation of precursor metabolites and energy
regulation of T cell activation
glycolytic process
ATP generation from ADP
heterotypic cell-cell adhesion
ADP metabolic process
purine nucleoside diphosphate metabolic process
purine ribonucleoside diphosphate metabolic process
nucleoside diphosphate phosphorylation
nucleotide phosphorylation
ribonucleoside diphosphate metabolic process
maintenance of location
T cell mediated cytotoxicity
sequestering of actin monomers
nucleoside diphosphate metabolic process
leukocyte cell-cell adhesion
maintenance of location in cell
Downregulated genes
M
Gzmb
Ly6e
Cxcr6
Id2
Ifngr1
Ccr2
Isg15
Cd47
Ccl4
Ccl3
Ccl5
Ifng
Ifi27l2a
Nr4a2
Tox
Bcl2a1b
Ifng high effector-like CD8+ T_d8C
Exhausted CD8+ T_d8C

Figure.S4. (A) UMAP projection within each point in time, color-coded by major cell lineages and transcript counts. (B) Heatmap showing mean expression (log (TPM+1)) of marker genes for the identified cell clusters. Cell cluster IDs on the left correspond to those in Fig. 4 A. (C) Percentage of each identified cell subtype Ifng high effector-like $CD8^+$ T across groups during Arm and Cl13 infections. (D) Flow cytometry of Ifng high effector-like $CD8^+$ T cells in lymph nodes of mice infected with the virus on the 4 and 8 days. (E and F) Violin plots showing the differentially expressed genes Gzma high effector-like $CD8^+$ T and Ifng high effector-like $CD8^+$ T about D8 Cl13 vs. Arm, respectively. (G and H) Violin plots showing the differentially expressed genes Gzma high effector-like $CD8^+$ T and Ifng high effector-like $CD8^+$ T from Cl13 infection group and Arm infection group on day 8, respectively. (I and J) The heatmap shows the differential expression of relevant genes for Gzma high effector-like $CD8^+$ T and Ifng high effector-like $CD8^+$ T from day 8, respectively. (K and L) GO BP enrichment analysis of differentially expressed genes upregulated about D8 Cl13 vs. Arm and downregulated about D4 Cl13 vs. Arm in Gzma high effector-like $CD8^+$ T. (M and N) GO BP enrichment analysis of differentially expressed genes upregulated and downregulated in Ifng high effector-like $CD8^+$ T (D8 Arm vs. Cl13). The top 20 enriched GO terms are shown. GO rich column-shaped diagram. The vertical coordinates are rich GO terms, and the horizontal coordinates are the number of different genes in the term. (O) Violin plots showing the differentially expressed genes Ifng high effector-like $CD8^+$ T vs. Exhausted $CD8^+$ T from day 8 Cl13.

Figure.S5

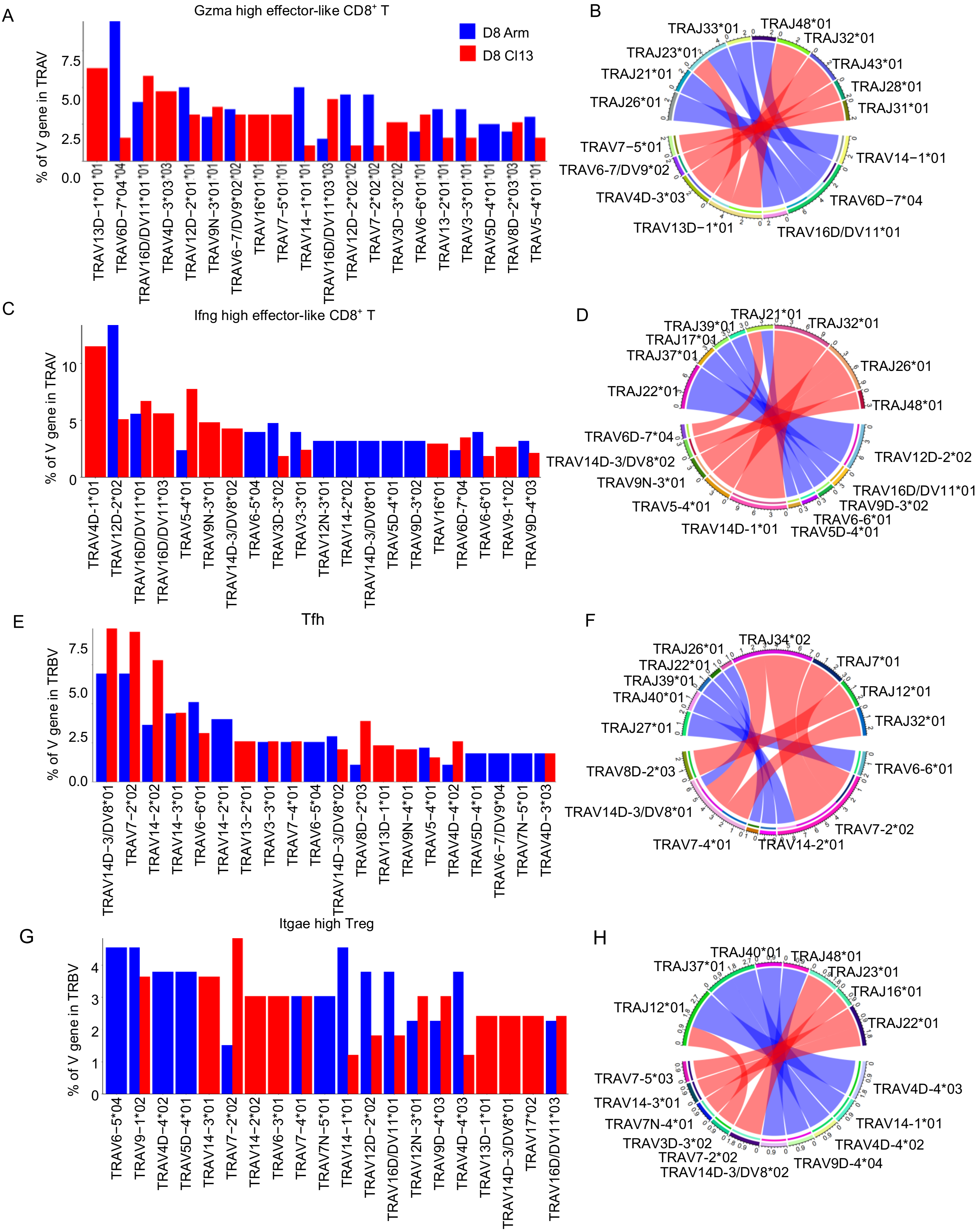

A
Gzma high effector-like CD8+ T
D8 Arm
D8 Cl13
% of V gene in TRAV
0.0
2.5
5.0
7.5
TRAV13D−1*01
TRAV6D−7*04
TRAV16D/DV11*01
TRAV4D−3*03
TRAV12D−2*01
TRAV9N−3*01
TRAV6−7/DV9*02
TRAV16*01
TRAV7−5*01
TRAV14−1*01
TRAV16D/DV11*03
TRAV12D−2*02
TRAV7−2*02
TRAV3D−3*02
TRAV6−6*01
TRAV13−2*01
TRAV3−3*01
TRAV5D−4*01
TRAV8D−2*03
TRAV5−4*01
B
TRAJ33*01
TRAJ48*01
TRAJ32*01
TRAJ23*01
TRAJ43*01
TRAJ21*01
TRAJ28*01
TRAJ26*01
TRAJ31*01
TRAV7−5*01
TRAV14−1*01
TRAV6-7/DV9*02
TRAV4D-3*03
TRAV6D−7*04
TRAV13D−1*01
TRAV16D/DV11*01
C
Ifng high effector-like CD8+ T
% of V gene in TRAV
0
5
10
TRAV4D-1*01
TRAV12D-2*02
TRAV16D/DV11*01
TRAV16D/DV11*03
TRAV5-4*01
TRAV9N-3*01
TRAV14D-3/DV8*02
TRAV6-5*04
TRAV3D-3*02
TRAV3-3*01
TRAV12N-3*01
TRAV14-2*02
TRAV14D-3/DV8*01
TRAV5D-4*01
TRAV9D-3*02
TRAV16*01
TRAV6D-7*04
TRAV6-6*01
TRAV9-1*02
TRAV9D-4*03
D
TRAJ21*01
TRAJ39*01
TRAJ32*01
TRAJ17*01
TRAJ37*01
TRAJ26*01
TRAJ22*01
TRAJ48*01
TRAV6D-7*04
TRAV12D-2*02
TRAV14D-3/DV8*02
TRAV9N-3*01
TRAV16D/DV11*01
TRAV5-4*01
TRAV9D-3*02
TRAV6-6*01
TRAV14D-1*01
TRAV5D-4*01
E
Tfh
% of V gene in TRBV
0.0
2.5
5.0
7.5
TRAV14D−3/DV8*01
TRAV7−2*02
TRAV14−2*02
TRAV14−3*01
TRAV6−6*01
TRAV14−2*01
TRAV13−2*01
TRAV3−3*01
TRAV7−4*01
TRAV6−5*04
TRAV14D−3/DV8*02
TRAV8D−2*03
TRAV13D−1*01
TRAV9N−4*01
TRAV5−4*01
TRAV4D−4*02
TRAV5D−4*01
TRAV6−7/DV9*04
TRAV7N−5*01
TRAV4D−3*03
F
TRAJ26*01
TRAJ34*02
TRAJ22*01
TRAJ7*01
TRAJ39*01
TRAJ12*01
TRAJ40*01
TRAJ32*01
TRAJ27*01
TRAV6-6*01
TRAV8D-2*03
TRAV14D-3/DV8*01
TRAV7-2*02
TRAV7-4*01
TRAV14-2*01
G
Itgae high Treg
% of V gene in TRBV
0
1
2
3
4
TRAV6−5*04
TRAV9−1*02
TRAV4D−4*02
TRAV5D−4*01
TRAV14−3*01
TRAV7−2*02
TRAV14−2*02
TRAV6−3*01
TRAV7−4*01
TRAV7N−5*01
TRAV14−1*01
TRAV12D−2*02
TRAV16D/DV11*01
TRAV12N−3*01
TRAV9D−4*03
TRAV4D−4*03
TRAV13D−1*01
TRAV14D−3/DV8*01
TRAV17*02
TRAV16D/DV11*03
H
TRAJ40*01
TRAJ48*01
TRAJ37*01
TRAJ23*01
TRAJ16*01
TRAJ12*01
TRAJ22*01
TRAV7-5*03
TRAV4D-4*03
TRAV14-3*01
TRAV14-1*01
TRAV7N-4*01
TRAV3D-3*02
TRAV4D-4*02
TRAV7-2*02
TRAV9D-4*04
TRAV14D-3/DV8*02

Figure S5. The clonal diversity of T repertoire revealed by scTCR-seq during acute and chronic virus infections. (A) The bar plots showed the usage of some V genes from Gzma high effector-like CD8$^+$ T cells TCR α chain of Arm group (blue)and Cl13 group (red) (8 dpi) (Top20). (B) Circos plots showed the differential TCR α VJ pairs in Gzma high effector-like CD8$^+$ T cells from the Arm and Cl13 groups. Blue links represent the acute group's specific VJ pairs, and red links represent the chronic group's specific VJ pairs. (C) The bar plots showed the usage of some V genes from Ifng high effector-like CD8$^+$ T cells TCR α chain of Arm group (blue)and Cl13 group (red) (8 dpi) (Top20). (D) Circos plots showed the differential TCR α VJ pairs in Ifng high effector-like CD8$^+$ T cells from the Arm and Cl13 groups. Blue links represent the acute group's specific VJ pairs, and red links represent the chronic group's specific VJ pairs. (E) The bar plots showed the usage of some V genes from Tfh cells TCR α chain of Arm group (blue)and Cl13 group (red) (8 dpi) (Top20). (F) Circos plots show the differential TCR α VJ pairs in Tfh cells from the Arm and Cl13 groups. Blue links represent the acute group's specific VJ pairs, and red links represent the chronic group's specific VJ pairs. (G) The bar plots showed the usage of some V genes from Itgae high Treg cells TCR α chain of Arm group (blue)and Cl13 group (red) (8 dpi) (Top20). (H) Circos plots show the differential TCR α VJ pairs in Itgae high Treg cells from the Arm and Cl13 groups. Blue links represent the acute group's specific VJ pairs, and red links represent the chronic group's specific VJ pairs.
